# Same-day initiation of oral pre-exposure prophylaxis among gay, bisexual, and other cisgender men who have sex with men and transgender women in Brazil, Mexico, and Peru (ImPrEP): a prospective, single-arm, open-label, multicentre implementation study

**DOI:** 10.1016/S2352-3018(22)00331-9

**Published:** 2022-12-21

**Authors:** Valdiléa G Veloso, Carlos F Cáceres, Brenda Hoagland, Ronaldo I Moreira, Hamid Vega-Ramírez, Kelika A Konda, Iuri C Leite, Sergio Bautista-Arredondo, Marcus Vinícius de Lacerda, José Valdez Madruga, Alessandro Farias, Josué N Lima, Ronaldo Zonta, Lilian Lauria, Cesar Vidal Osco Tamayo, Hector Javier Salvatierra Flores, Yovanna Margot Cabrera Santa Cruz, Ricardo Martín Moreno Aguayo, Marcelo Cunha, Júlio Moreira, Alessandra Ramos Makkeda, Steven Díaz, Juan V Guanira, Heleen Vermandere, Marcos Benedetti, Heather L Ingold, M Cristina Pimenta, Thiago S Torres, Beatriz Grinsztejn, ImPrEP Study Group, J. David Urbaez-Brito, Polyana d'Albuquerque, Claudio Palombo, Paulo Ricardo Alencastro, Raquel Keiko de Luca Ito, João L. Benedetti, Fabio V. Maria, Paula M. Luz, Lucilene Freitas, Kim Geraldo, Monica Derrico, Sandro Nazer, Tania Kristic, Renato Girade, Renato Lima, Antônio R. Carvalho, Carla Rocha, Pedro Leite, Marcio Lessa, Marilia Santini-Oliveira, Daniel R.B. Bezerra, Cleo de Oliveira Souza, Jacinto Corrêa, Marcelo Alves, Carolina Souza, Camilla Portugal, Mônica dos Santos Valões, Gabriel Lima Mota, Joyce Alves Gomes, Cynthia Ferreira Lima Falcão, Fernanda Falcão Riberson, Luciano Melo, Talita Andrade Oliveira, Agnaldo Moreira Oliveira Júnior, Bruna Fonseca, Leonor Henriette Lannoy, Ludymilla Anderson Santiago Carlos, João Paulo Cunha, Sonia Maria de Alencastro Coracini, Thiago Oliveira Rodrigues, Emília Regina Scharf Mettrau, Kelly Vieira Meira, Heder Tavares, Ana Paula Nunes Viveiros Valeiras, Taiane Miyake Alves de Carvalho Rocha, Alex Amorim, Patrícia Sabadini, Luiz Gustavo Córdoba, Caio Gusmão, Erika Faustino, Julia Soares da Silva Hansen, Agatha Mirian Cunha, Neuza Uchiyama Nishimura, Jaime Eduardo Flygare Razo Prereira Santos, Aline Barnabé Cano, Willyam Magnum Telles Dias, Magô Tonhon, Tania Regina Rezende, Alex Gomes, Eloá dos Santos Rodrigues, Maria das Dores Aires Carneiro, Alexandre Castilho, Mariana Carvalho, Dulce Diaz-Sosa, Centli Guillen-Diaz-Barriga, Lorena Hernández, Rebeca Robles, Maria Elena Medina-Mora, Marcela González, Ivonne Huerta Icelo, Araczy Martinez Davalos, José Gomez Castro, Luis Obed Ocampo Valdez, Fernanda Ramírez Barajas, Verónica Ruiz González, Galileo Vargas Guadarrama, Israel Macías, Jehovani Tena Sánchez, Juan Pablo Osuna Noriega, H. Rodrigo Moheno M, Jorge M. Bernal Ramírez, Víctor Dante Galicia Juarez, Gerardo Vizcaíno, Francisco Javier Arjona, Gino Calvo, Silver Vargas, Oliver Elorreaga, Ximena Gutierrez, Fernando Olivos, Damaris Caviedes, Daniella Adriazola, Eduardo Juárez, Gabriela Mariño, Jazmin Qquellon, Francesca Vasquez, Jean Pierre Jiron, Sonia Flores, Karen Campos

**Affiliations:** a Instituto Nacional de Infectologia Evandro Chagas, Fundação Oswaldo Cruz (INI-Fiocruz), Rio de Janeiro, Brazil; b Universidad Peruana Cayetano Heredia, Centro de Investigaciones Interdisciplinarias em Salud, Sexualidad, y SIDA, Lima, Peru; c Instituto Nacional de Psiquiatria Ramón de la Fuente Muñiz, Mexico City, Mexico; d Escola Nacional de Saúde Pública, Fundação Oswaldo Cruz (ENSP-Fiocruz), Rio de Janeiro, Brazil; e Instituto Nacional de Salud Pública (INSP), Cuernavaca, Mexico; f Fundação de Medicina Tropical Heitor Vieira Dourado, Manaus, Brazil; g Centro de Referência e Treinamento em DST/AIDS—CRT-SP, São Paulo, Brazil; h CEDAP—Centro Estadual Especializado em Diagnóstico, Assistência e Pesquisa, Salvador, Brazil; i Centro de Referência em DST/AIDS—AMDA, Campinas, Brazil; j Policlínica Centro, Florianópolis, Brazil; k Hospital Municipal Rocha Maia, Rio de Janeiro, Brazil; l Centro de Referencia de Infecciones de Transmisión Sexual del Centro Materno Infantil Tahuantinsuyo Bajo, Lima, Peru; m Centro de Referencia de Infecciones de Transmisión Sexual del Centro de Salud Alberto Barton, Callao, Peru; n Centro de Referencia de Infecciones de Transmisión Sexual de Caja de Agua, Lima, Peru; o Centro de Referencia de Infecciones de Transmisión Sexual del Hospital Amazónico Pucallpa, Ucayali, Peru; p United Nations Population Fund, Mexico City, Mexico; q UNITAID, Geneva, Switzerland; r Ministério da Saúde, Brasília, Brazil

## Abstract

**Background:**

Although gay, bisexual, and other cisgender men who have sex with men (MSM) and transgender women have the highest HIV burden in Latin America, pre-exposure prophylaxis (PrEP) implementation is poor. We aimed to assess the feasibility of same-day oral PrEP delivery in Brazil, Mexico, and Peru.

**Methods:**

Implementation PrEP (ImPrEP) was a prospective, single-arm, open-label, multicentre PrEP implementation study conducted in Brazil (14 sites), Mexico (four sites), and Peru (ten sites). MSM and transgender women were eligible to participate if they were aged 18 years or older, HIV-negative, and reported one or more prespecified criteria. Enrolled participants received same-day initiation of daily oral PrEP (tenofovir disoproxil fumarate [300 mg] coformulated with emtricitabine [200 mg]). Follow-up visits were scheduled at week 4 and quarterly thereafter. We used logistic regression models to identify factors associated with early loss to follow-up (not returning after enrolment), PrEP adherence (medication possession ratio ≥0·6), and long-term PrEP engagement (attending three or more visits within 52 weeks). This study is registered at the Brazilian Registry of Clinical Trials, U1111-1217-6021.

**Findings:**

From Feb 6, 2018, to June 30, 2021, 9979 participants were screened and 9509 were enrolled (Brazil n=3928, Mexico n=3288, and Peru n=2293). 543 (5·7%) participants were transgender women, 8966 (94·3%) were cisgender men, and 2481 (26·1%) were aged 18–24 years. There were 12 185·25 person-years of follow-up. 795 (8·4%) of 9509 participants had early loss to follow-up, 6477 (68·1%) of 9509 were adherent to PrEP, and 5783 (70·3%) of 8225 had long-term PrEP engagement. Transgender women (adjusted odds ratio 1·60, 95% CI 1·20–2·14), participants aged 18–24 years (1·80, 1·49–2·18), and participants with primary education (2·18, 1·29–3·68) had increased odds of early loss to follow-up. Transgender women (0·56, 0·46–0·70), participants aged 18–24 years (0·52, 0·46–0·58), and those with primary education (0·60, 0·40–0·91) had lower odds of PrEP adherence. Transgender women (0·56, 0·45–0·71), participants aged 18–24 years (0·56, 0·49–0·64), and those with secondary education (0·74, 0·68–0·86) had lower odds of long-term PrEP engagement. HIV incidence was 0·85 per 100 person-years (95% CI 0·70–1·03) and was higher for transgender women, participants from Peru, those aged 18–24 years, Black and mixed-race participants, and participants who were non-adherent to PrEP.

**Interpretation:**

Same-day oral PrEP is feasible for MSM and transgender women in Latin America. Social and structural determinants of HIV vulnerability need to be addressed to fully achieve the benefits of PrEP.

**Funding:**

Unitaid, WHO, and Ministries of Health in Brazil, Mexico, and Peru.

**Translations:**

For the Portuguese and Spanish translations of the abstract see Supplementary Materials section.

## Introduction

Tenofovir-based oral pre-exposure prophylaxis (PrEP) has dramatically reduced population-level HIV incidence in London (UK),^1^ San Francisco (CA, USA),^2^ New South Wales (Australia),^3^ and in rural Kenya and Uganda.^4^ Access to PrEP can reduce HIV incidence among groups disproportionately affected by HIV, including youth, gay, bisexual, and other cisgender men who have sex with men (MSM), cisgender women, and transgender women.^5^

**Research in context**

**Evidence before this study**

We searched PubMed using the terms (“PrEP” OR “pre-exposure prophylaxis” OR “combination prevention”) AND (“HIV” OR “human immunodeficiency virus”) AND (“Brazil” OR “Mexico” OR “Peru” OR “Latin America”) on Sept 11, 2022, with no restriction of publication date or language, in addition to reviewing reference lists of relevant articles. We reviewed the literature on research pertaining to pre-exposure prophylaxis (PrEP) services among gay, bisexual, and other cisgender men who have sex with men (MSM) and transgender women in Latin America, with a particular focus on same-day oral PrEP and PrEP outcomes such as adherence and persistence. PrEP still has poor availability across much of Latin America. Availability of PrEP is a public health policy in Brazil through the Brazilian National Public Health System (Sistema Único de Saúde) since December 2017, the Latin American country in which most PrEP research and implementation has taken place. Mexico has offered PrEP through the public health system since mid-2021, and PrEP is a component of a new public health policy still awaiting approval in Peru. We found results reported only for three PrEP demonstration studies in Brazil (PrEP Brasil study [n=450; Rio de Janeiro and São Paulo], PrEParadas [n=130; Rio de Janeiro], and a study conducted in Recife [n=219]).

**Added value of this study**

To our knowledge, the Implementation PrEP (ImPrEP) study is the largest PrEP implementation study in Latin America. We aimed to evaluate the feasibility of PrEP implementation among MSM and transgender women in the context of the public health systems of Brazil, Mexico, and Peru. Our findings show that a same-day daily oral PrEP delivery strategy was feasible and effective, with only a small proportion of participants with early loss to follow-up. HIV incidence during the study was low, and most participants had PrEP adherence and long-term PrEP engagement, although country, sociodemographic, and behavioural disparities were observed. Participants from Peru, transgender women, younger participants (aged 18–24 years), and individuals who were not White had higher HIV incidence, increased odds of early loss to follow-up, and lower odds of PrEP adherence and long-term PrEP engagement. We observed high incidence of sexually transmitted infections overall, and higher incidence among those of younger age and transgender women.

**Implications of all the available evidence**

PrEP services in Latin America should focus on strategies to encourage PrEP reinitiation, adherence, and persistence among populations highly vulnerable to HIV infection. Social and structural determinants of HIV vulnerability need to be addressed to fully realise PrEP benefits. Our results support and inform PrEP programmes in Latin America and other low-income and middle-income regions. Gender-affirming services for transgender populations and strategies to engage and retain young individuals might improve PrEP outcomes. Long-acting PrEP strategies might overcome these identified pitfalls.

Initiation and continuation of PrEP during periods of higher susceptibility to HIV are required to realise its full benefits. Reasons for stopping or never initiating PrEP include, in addition to access, low self-perceived risk, concerns about side-effects or a desire not to take a daily pill, stigma, or competing life events.^6^, ^7^ Furthermore, linkage from HIV testing to a PrEP prescription appointment occurs only for 21–67% of individuals referred for PrEP care.^4^, ^8^ To address this gap, PrEP programmes focus on scaling up PrEP uptake, especially among the most clinically vulnerable^8^ (eg, by implementing same-day PrEP, in which a prescription is provided the day that PrEP eligibility is confirmed).^9^

In 2021, 2·2 million people were living with HIV in Latin America. New HIV infections in the region are estimated to have increased in the past decade, with most new cases among MSM and transgender women.^5^ HIV prevalence in the region was estimated at 13·9% for MSM (2016–20) and 25·9% for transgender women (meta-analysis), with differences among and within countries.^5^, ^10^, ^11^ Key populations in Latin America are at increased clinical vulnerability due to inadequate access to HIV services, stigma, discrimination, and other human rights violations.^5^

Although daily oral PrEP was recommended for people at substantial HIV risk by WHO in 2014, PrEP availability is limited in Latin America. Only 10% of PrEP users across 77 countries are in Latin America, most of whom are in Brazil.^12^ PrEP has been available within Brazil's Public Health System (Sistema Único de Saúde) since December, 2017, has been recently implemented as a public health policy in Mexico, and is part of a new policy awaiting approval in Peru, where it remains accessible only via purchase or through a few research studies.

To increase the body of evidence on PrEP implementation in Latin America, we report here the results of the Implementation PrEP (ImPrEP) study. We aimed to assess the feasibility of same-day oral PrEP delivery in Brazil, Mexico, and Peru, including factors associated with early loss to follow-up, PrEP adherence, long-term PrEP engagement, HIV incidence, and the prevalence and incidence of sexually transmitted infections during follow-up.

## Methods

### Study design and participants

ImPrEP was a prospective, single-arm, open-label, multicentre PrEP implementation study that enrolled a convenience sample of participants in Brazil (at 14 HIV and sexually transmitted infection clinics in 11 cities), Mexico (at one HIV and sexually transmitted infection clinic and three community organisations in three cities), and Peru (at ten HIV and sexually transmitted infection clinics in six cities).

Eligible participants were HIV-negative cisgender MSM and transgender women, aged 18 years and older, and reporting one or more of the following criteria in the previous 6 months (following Brazilian PrEP Guidelines): condomless anal sex, anal sex with partner or partners living with HIV, reported rectal or urethral gonorrhoea, reported rectal or urethral chlamydia, reported syphilis, or transactional sex.

Institutional review boards at Instituto Nacional de Infectologia Evandro Chagas, Fundação Oswaldo Cruz (Rio de Janeiro, Brazil; approval number CAAE 79259517.5.1001.5262), National Institute of Public Health (Mexico City, Mexico; approval number CI-1515), and Universidad Peruana Cayetano Heredia (Lima, Peru; approval number 100740) approved the study. Ethics approvals were also obtained for WHO Research Ethics Review Committee and local institutional review boards at each Brazilian site. All study participants provided written informed consent before enrolment in Portuguese (Brazil) or Spanish (Mexico and Peru). The study was done according to the Declaration of Helsinki principles.

### Procedures

Participants were recruited through social media advertisements, peer and health-care provider referrals, and by community education teams of MSM and transgender women peer-educators. Potentially eligible individuals were screened using HIV rapid tests (Brazil: ABON HIV1/2/O Tri-line, Abon Biopharm, Hangzhou, China; Mexico and Peru: Alere Determine HIV-1/2 Ag/Ab Combo, Abbott, Chiba, Japan) and clinical and behavioural criteria and, if eligible, participants were enrolled to receive same-day daily oral PrEP with tenofovir disoproxil fumarate (300 mg) coformulated with emtricitabine (200 mg). We provided Truvada (Gilead Sciences, Martinsried, Germany) in Brazil and Mexico, and generic tenofovir disoproxil fumarate plus emtricitabine (Brazil: Blanver, São Paulo, Brazil; Mexico: Sanofi Movitrem, Sandoz, Taihou, China; Peru: Mylan Lab, Munbai, India) in Brazil, Mexico, and Peru.

HIV viral load testing was done for all individuals with a non-reactive HIV rapid test at enrolment (Brazil and Mexico: Abbott RealTime HIV-1 m2000, Abbott, Wiesbaden, Germany; Peru: Hologic Aptima HIV-1 RNA Qualitative assay, San Diego, CA, USA). Participants diagnosed with chronic or acute HIV infection (negative antibody rapid test and detectable viral load) had their antiretroviral therapy regimen optimised according to the country's standard of care. Serum creatinine was evaluated at enrolment. HIV viral load and serum creatinine test results were not provided on the same day. Participants were contacted to repeat the serum creatinine clearance test if the result was 60 mL/min or less. Data on demographics, sexual behaviour, substance use, as well as the main reason for attending the service were recorded at enrolment. Clinical staff provided HIV and sexually transmitted infection prevention counselling at all study visits. Condoms and lubricants were provided.

Follow-up visits were scheduled at week 4 and quarterly thereafter until study termination (June 30, 2021). At each visit, participants received tenofovir disoproxil fumarate plus emtricitabine refills according to the next scheduled visit interval. Individuals who returned more than 24 weeks after any visit were required to re-enrol and undergo the same visit procedures performed at enrolment. HIV testing was done at all study visits using rapid tests. HIV confirmatory testing was conducted according to each country's algorithm. Self-reported adherence was assessed at week 4 and quarterly visits. Adverse events were assessed at each visit.

All sexually transmitted infection examinations were done at enrolment. Syphilis testing was done at enrolment and at quarterly visits with a rapid *Treponema pallidum* test and positive results were confirmed using non-treponemal tests (venereal disease research laboratory in Brazil and Mexico; rapid plasma reagin in Peru). Active or recent syphilis at enrolment was defined as titres of 1:8 or more and a positive microhaemagglutination assay for *T pallidum*. Incident syphilis infections during follow-up were recorded if no previous infection was reported or if the participant had adequate treatment for a previous syphilis diagnosis and had a four-times increase in titre, and this titre was at least 1:8. Self-collected rectal swabs for the detection of chlamydia (*Chlamydia trachomatis*) and gonorrhoea (*Neisseria gonorrhoeae*) were done at enrolment and on an annual basis; in Brazil, the Abbott Real Time platform and the CT/NG Amplification Reagent Kit (Abbott Molecular, Des Plains, IL, USA) were used; in Mexico, STD Direct Flow chip Máster Diagnóstica, Granada, Spain; and in Peru, Hologic Aptima Combo 2 assay, San Diego, CA, USA. Hepatitis B and hepatitis C testing were done at enrolment and on an annual basis using HBsAg and anti-hepatitis C virus rapid tests. Individuals with a negative hepatitis B test at enrolment and reporting no previous vaccination were referred for vaccination where available. All sexually transmitted infection results were available before the next study visit. Treatment for sexually transmitted infections was provided at all study sites according to local standard of care.

Gender of participants was dichotomised into cisgender man and transgender woman. Age at enrolment was described as median (IQR), and in categorical ranges. We categorised self-reported race as Asian, Black, Indigenous, mixed race (*pardo* or *mestizo*), or White. Education was collected differently by country to capture local educational strata and then recategorised for analyses into primary (complete or incomplete), secondary (complete or incomplete), and more than secondary. The main reason to attend the service was stratified as seeking PrEP or other (seeking an HIV test, other health service, or postexposure prophylaxis). Postexposure prophylaxis use in the 12 months before enrolment was dichotomised as yes or no.

Sexual behaviour was assessed with questions related to number of cisgender men or transgender women sex partners (described with median and IQR, categorised into <5, 5–10, or >10 for analyses), receptive condomless anal sex (yes or no), condomless anal sex with a partner or partners living with HIV (yes, no, or I don't know), and transactional sex (sex in exchange for money, drugs, gifts, or favours; yes or no). Binge drinking was assessed with “Did you have five or more drinks within a two-hour period?” (yes or no). Stimulant use was defined as use of club drugs (eg, ecstasy, lysergic acid diethylamide, or gamma-hydroxybutyrate), cocaine (powder, crack, or paste), poppers, or other inhalants. At enrolment, questions on sexual behaviour and substance use referred to the previous 6 months, except for number of sex partners in Brazil and Mexico, which referred to the previous 3 months. Self-reported adherence was assessed with “In the previous 30 days, approximately how many pills did you NOT take?” Individuals who reported not missing any pill during the previous 30 days were deemed adherent as described.^13^

### Outcomes

Four study outcomes were evaluated. The coprimary outcomes were PrEP adherence and duration of PrEP measured as early loss to follow-up and long-term PrEP engagement. PrEP adherence was defined as having a medication possession ratio (MPR) of 0·6 or more, equivalent to four tenofovir disoproxil fumarate plus emtricitabine pills weekly and enough for highly protective levels of tenofovir diphosphate.^14^ MPR was calculated by dividing the total number of pills dispensed by total number of days between enrolment and the last attended visit. Early loss to follow-up was defined as attending the enrolment visit and not returning to any follow-up visit. Long-term PrEP engagement was defined as attending the week 4 visit plus two or more quarterly visits within a 52-week follow-up period. Secondary outcomes were the incidence of HIV and syphilis and the prevalence of sexually transmitted infections. We defined HIV incidence as any HIV infection detected after enrolment. We estimated the prevalence of syphilis at enrolment and the incidence of syphilis during follow-up. Prevalence of rectal chlamydia, rectal gonorrhoea, hepatitis B, and hepatitis C were assessed at enrolment and at week 52.

### Statistical analysis

Characteristics of all enrolled participants and enrolment frequency were recorded overall and per country (Brazil, Mexico, and Peru). We carried out three analyses using logistic regression models to identify factors associated with early loss to follow-up among all enrolled participants, factors associated with PrEP adherence among all enrolled participants, and factors associated with long-term PrEP engagement among participants who had time to complete 52 weeks of follow-up. Sociodemographic and behavioural characteristics, measured at enrolment, were included as potential predictors: country, gender, age group, race (dichotomised as White and Black, mixed race, Indigenous, or Asian), education, main reason to attend the service, number of cisgender men or transgender women sex partners, receptive condomless anal sex, condomless anal sex with a partner or partners living with HIV, transactional sex, and substance use. For long-term PrEP engagement, we also included self-reported adherence at week 4, with individuals who did not return for follow-up visits after enrolment considered as non-adherent. In all univariable models, the effect of each variable was controlled for country and all statistically significant variables at a p value of 0·1 or less were included in the final multivariable model. Country-specific model results were calculated for each outcome.

Incidence of HIV was calculated as the number of HIV incident cases during the person-years of follow-up, presented per 100 person-years overall and stratified by country, gender, age, race, and adherence (MPR). Incidence of syphilis was calculated as the number of incident cases during the person-years of follow-up, presented per 100 person-years. We estimated incidence and 95% CI using a Poison regression model, and calculated person-years under follow-up considering time between enrolment and the last clinical visit before June 30, 2021. For prevalence of rectal chlamydia, rectal gonorrhoea, hepatitis B, and hepatitis C we estimated the 95% CI using normal approximations or exact estimation in the case of sparse cells. We evaluated differences in prevalence between enrolment and week 52 using generalised estimating equations logistic models and when the number of events was small, we used exact logistic models. Sexually transmitted infection results were presented overall and stratified by country, gender, and age. All analyses were performed using SAS version 9.4.

This study is registered at the Brazilian Registry of Clinical Trials, U1111-1217-6021.

### Role of the funding source

The funders of the study had no role in study design, data collection, data analysis, data interpretation, or writing of the report.

## Results

From Feb 6, 2018, to June 30, 2021, 9979 participants were screened and 9509 were enrolled (Brazil n=3928, Mexico n=3288, and Peru n=2293; appendix 3 p 2). Participants were followed up until June 30, 2021. 43 patients had acute HIV infection (HIV-negative rapid test or detectable HIV viral load) identified at enrolment and were subsequently referred to HIV care. Thus, the prevalence of acute infection at the screening visit was 0·5%. 915 (9·6%) participants returned more than 24 weeks after a previous visit and were thus re-enrolled in the study (appendix 3 p 3). Re-enrolments were more common in Peru (618; 26·9%) than in Brazil (232; 5·9%) and Mexico (65; 2·0%).

Among the 9509 enrolled participants, median age was 29 years (IQR 24–35); 2481 (26·1%) were aged 18–24 years and 5984 (62·9%) self-identified as mixed race (table 1). The majority had more than secondary education, had attended the clinic seeking PrEP, and reported no postexposure prophylaxis use in the previous 12 months (table 1). Sexual behaviour, binge drinking, and stimulant use are shown in table 1. Based on self-reported information, 5235 (55·1%) participants did not report missing any pill during the previous 30 days and were thus deemed adherent at week 4. 60 participants (0·63%, 95% CI 0·47–0·79) with creatinine clearance 60 mL/min or less at enrolment had repeat creatinine testing, and subsequent results were within the permitted range.

**Table 1:** Participant characteristics at enrolment

	**Total (n=9509)**	**Brazil (n=3928)**	**Mexico (n=3288)**	**Peru (n=2293)**
**Gender**				
Cisgender man	8966 (94·3%)	3733 (95·0%)	3193 (97·1%)	2040 (89·0%)
Transgender woman	543 (5·7%)	195 (5·0%)	95 (2·9%)	253 (11·0%)
**Age, years**				
Median (IQR)	29 (24–35)	29 (24–35)	30 (25–35)	27 (23–33)
18–19	349 (3·7%)	122 (3·1%)	54 (1·6%)	173 (7·5%)
20–21	670 (7·0%)	269 (6·8%)	176 (5·4%)	225 (9·8%)
22–24	1462 (15·4%)	642 (16·3%)	413 (12·6%)	407 (17·7%)
25–30	3067 (32·3%)	1216 (31·0%)	1156 (35·2%)	695 (30·3%)
>30	3961 (41·7%)	1679 (42·7%)	1489 (45·3%)	793 (34·6%)
**Race**				
Asian	63 (0·7%)	42 (1·1%)	12 (0·4%)	9 (0·4%)
Black	813 (8·5%)	595 (15·1%)	45 (1·4%)	173 (7·5%)
Indigenous	95 (1·0%)	13 (0·3%)	63 (1·9%)	19 (0·8%)
Mixed race*	5984 (62·9%)	1410 (35·9%)	2715 (82·6%)	1859 (81·1%)
White	2554 (26·9%)	1868 (47·6%)	453 (13·8%)	233 (10·2%)
**Education**				
Primary, complete or incomplete	113 (1·2%)	52 (1·3%)	23 (0·7%)	38 (1·7%)
Secondary, complete or incomplete	1662 (17·5%)	775 (19·7%)	182 (5·5%)	705 (30·8%)
More than secondary	7734 (81·3%)	3101 (78·9%)	3083 (93·8%)	1550 (67·6%)
**Main reason to attend the service**				
Seeking PrEP	8348 (87·8%)	3774 (96·1%)	3126 (95·1%)	1448 (63·1%)
Other	1161 (12·2%)	154 (3·9%)	162 (4·9%)	845 (36·9%)
**Postexposure prophylaxis use**†				
Yes	1692 (17·8%)	1119 (28·5%)	510 (15·5%)	63 (2·7%)
No	7817 (82·2%)	2809 (71·5%)	2778 (84·5%)	2230 (97·3%)
**Number of sex partners**‡				
Median (IQR)	5 (3–15)	5 (2–15)	8 (4–15)	4 (2–10)
<5	4011 (42·2%)	1813 (46·2%)	1045 (31·8%)	1153 (50·3%)
5–10	2683 (28·2%)	968 (24·6%)	1107 (33·7%)	608 (26·5%)
>10	2815 (29·6%)	1147 (29·2%)	1136 (34·5%)	532 (23·2)
**Receptive condomless anal sex**§				
Yes	6252 (65·7%)	2557 (65·1%)	2340 (71·2%)	1355 (59·1%)
No	3257 (34·3%)	1371 (34·9%)	948 (28·8%)	938 (40·9%)
**Condomless anal sex with partner or partners living with HIV**§				
Yes	1892 (19·9%)	824 (21·0%)	831 (25·3%)	237 (10·3%)
No	2640 (27·8%)	1144 (29·1%)	752 (22·9%)	744 (32·4%)
I don't know	4976 (52·3%)	1960 (49·9%)	1704 (51·8%)	1312 (57·2%)
**Transactional sex**§				
Yes	1643 (17·3%)	397 (10·1%)	732 (22·3%)	514 (22·4%)
No	7865 (82·7%)	3531 (89·9%)	2555 (77·7%)	1779 (77·6%)
**Binge drinking**§				
Yes	6254 (65·8%)	2566 (65·3%)	2009 (61·1%)	1679 (73·2%)
No	3254 (34·2%)	1362 (34·7%)	1278 (38·9%)	614 (26·8%)
**Stimulant use**§¶				
Yes	1743 (18·3%)	715 (18·2%)	888 (27·0%)	140 (6·1%)
No	7766 (81·7%)	3213 (81·8%)	2400 (73·0%)	2153 (93·9%)
**Self-reported PrEP adherence at week 4**‖				
Yes	5235 (55·1%)	2503 (63·7%)	2001 (60·9%)	731 (31·9%)
No	4274 (44·9%)	1425 (36·3%)	1287 (39·1%)	1562 (68·1%)

**Pr**EP=pre-exposure prophylaxis.

* For Brazil, mixed race refers to *pardo*; for Mexico and Peru, mixed race refers to *mestizo*.

† Past 12 months.

‡ For Brazil and Mexico: past 3 months; for Peru: past 6 months.

§ Past 6 months.

¶ Stimulant use was defined as use of any: club drugs (eg, ecstasy, lysergic acid diethylamide, and gamma-hydroxybutyrate), cocaine (powder, crack, or paste), poppers, or other inhalants.

‖ Report of no missing pill in the previous 30 days.

Overall, participants were followed up for 12 185·25 person-years, with more person-years of follow-up in Brazil than in Mexico or Peru (table 2). 104 HIV seroconversions occurred with an HIV incidence of 0·85 (95% CI 0·70–1·03) per 100 person-years. The incidence was greater in Peru than in Mexico or Brazil (table 2). HIV incidence was 2·5 times greater among transgender women than among MSM, 4·3 times greater among younger (aged 18–24 years) participants than older (aged >30 years) participants, and 2·2 times greater among participants who were Black, mixed race, Indigenous, or Asian than among participants who were White (table 2). HIV incidence among participants not adherent to PrEP (MPR <0·6) was 5·3 times greater than among participants adherent to PrEP (MPR ≥0·6), and differences were more pronounced when further stratified by country, gender, age, and race (table 2).

**Table 2:** HIV incidence overall and according to adherence (MPR) stratified per country, gender, age, and race

		**Overall**			**Non-adherence (MPR <0·6)**			**Adherence (MPR ≥0·6)**		
		HIV infections	Person-years of follow-up*	Incidence per 100 person-years (95% CI)	HIV infections	Person-years of follow-up	Incidence per 100 person-years (95% CI)	HIV infections	Person-years of follow-up	Incidence per 100 person-years (95% CI)
Overall		104	12 185·25	0·85 (0·70–1·03)	46	1584·59	2·90 (2·17–3·88)	58	10 600·67	0·55 (0·42–0·71)
Country										
	Brazil	24	6577·81	0·36 (0·24–0·54)	7	501·14	1·40 (0·67–2·93)	17	6076·67	0·28 (0·17–0·45)
	Mexico	18	3242·08	0·56 (0·35–0·88)	7	385·88	1·81 (0·86–3·81)	11	2856·21	0·39 (0·21–0·70)
	Peru	62	2365·36	2·62 (2·04–3·36)	32	697·57	4·59 (3·24–6·49)	30	1667·79	1·80 (1·26–2·57)
Gender										
	Cisgender man	93	11 625·85	0·80 (0·65–0·98)	41	1433·63	2·86 (2·11–3·88)	52	10192·21	0·51 (0·39–0·67)
	Transgender woman	11	559·41	1·97 (1·09–3·55)	5	150·95	3·31 (1·38–7·96)	6	408·45	1·47 (0·66–3·27)
Age, years										
	18–24	55	2827·88	1·94 (1·49–2·53)	27	521·12	5·18 (3·55–7·56)	28	2306·76	1·21 (0·84–1·76)
	25–30	24	3761·77	0·64 (0·43–0·95)	10	519·02	1·93 (1·04–3·58)	14	3242·75	0·43 (0·26–0·73)
	>30	25	5595·61	0·45 (0·30–0·66)	9	544·45	1·65 (0·86–3·18)	16	5051·15	0·32 (0·19–0·52)
Race										
	White	18	3862·60	0·47 (0·29–0·74)	7	348·38	2·01 (0·96–4·21)	11	3514·22	0·31 (0·17–0·57)
	Black, mixed race, Indigenous, or Asian	86	8322·65	1·03 (0·84–1·28)	39	1236·21	3·15 (2·31–4·32)	47	7086·44	0·66 (0·50–0·88)
Race in Brazil only										
	White	9	3170·24	0·28 (0·15–0·55)	2	199·47	1·00 (0·25–0·40)	7	2970·78	0·24 (0·11–0·49)
	Black, mixed race, Indigenous, or Asian	15	3407·57	0·44 (0·27–0·73)	5	301·67	1·66 (0·69–3·98)	10	3105·90	0·32 (0·17–0·60)

MPR=medication possession ratio.

* Median length of follow-up overall: 1·42 years (IQR 0·56–2·21); Brazil: 1·99 years (1·00–2·49); Mexico: 1·04 years (0·38–1·54); Peru: 1·19 years (0·42–1·94).

Among all enrolled participants, 795 (8·4%) never returned for any follow-up visit after enrolment and were considered as an early loss to follow-up (table 3). The proportion of participants recorded as early loss to follow-up was greater in Peru than in Mexico or Brazil (table 3). The odds of early loss to follow-up were greater among transgender women versus cisgender men, among participants aged 18–24 years and 25–30 years versus those older than 30 years, greater among participants with primary or secondary education versus those with more than secondary education, greater among participants reporting condomless anal sex with a partner or partners with unknown HIV status versus those reporting not taking part in condomless anal sex with a partner or partners with HIV, and greater among those reporting taking part in transactional sex versus those reporting not (table 3). The odds of early loss to follow-up were lower among participants who self-identified as White versus those of other races, lower among participants attending the service seeking PrEP versus those attending for other reasons, lower among participants reporting five to ten or more than ten sex partners versus those reporting fewer than five sex partners, and lower among participants reporting receptive condomless anal sex versus those reporting not taking part in this behaviour (table 3). Although similar results were observed in the models for each country, in Peru the following variables were not statistically significant: gender, race, and transactional sex. Non-significant variables were race in Mexico, and receptive condomless anal sex in Brazil (appendix 3 p 5).

**Table 3:** Factors associated with early loss to follow-up

	**Number of participants (n=9509)**	Early loss to follow-up*(n=795 [8·4%])	**Univariable analyses**		**Multivariable analysis**	
			OR (95% CI)	p value	aOR (95% CI)	p value
**Country**						
Brazil	3928	163 (4·2%)	1 (ref)	..	1 (ref)	..
Mexico	3288	259 (7·9%)	1·98 (1·61–2·42)	<0·0001	2·20 (1·76–2·74)	<0·0001
Peru	2293	373 (16·3%)	4·49 (3·70–5·44)	<0·0001	2·74 (2·10–3·42)	<0·0001
**Gender**						
Cisgender man	8966	702 (7·8%)	1 (ref)	..	1 (ref)	..
Transgender woman	543	93 (17·1%)	1·95 (1·53–2·49)	<0·0001	1·60 (1·20–2·14)	0·0015
**Age, years**						
18–24	2481	294 (11·8%)	1·91 (1·59–2·29)	<0·0001	1·80 (1·49–2·18)	<0·0001
25–30	3067	265 (8·6%)	1·44 (1·20–1·74)	<0·0001	1·52 (1·26–1·84)	<0·0001
>30	3961	236 (6·0%)	1 (ref)	..	1 (ref)	..
**Race**						
White	2554	119 (4·7%)	0·70 (0·56–0·87)	0·0011	0·75 (0·60–0·94)	0·011
Black, mixed race, Indigenous, or Asian	6955	676 (9·7%)	1 (ref)	..	1 (ref)	..
**Education**						
Primary, complete or incomplete	113	21 (18·6%)	3·05 (1·86–5·02)	<0·0001	2·18 (1·29–3·68)	0·0036
Secondary, complete or incomplete	1662	249 (15·0%)	2·18 (1·84–2·60)	<0·0001	1·76 (1·46–2·12)	<0·0001
More than secondary	7734	525 (6·8%)	1 (ref)	..	1 (ref)	..
**Main reason to attend the service**						
Seeking PrEP	8348	585 (7·0%)	0·57 (0·47–0·70)	<0·0001	0·64 (0·52–0·78)	<0·0001
Other	1161	210 (18·1%)	1 (ref)	..	1 (ref)	..
**Number of sex partners**†						
<5	4011	400 (10·0%)	1 (ref)	..	1 (ref)	..
5–10	2683	212 (7·9%)	0·78 (0·65–0·93)	0·0060	0·74 (0·61–0·89)	0·0017
>10	2815	183 (6·5%)	0·67 (0·56–0·81)	<0·0001	0·50 (0·40–0·62)	<0·0001
**Receptive condomless anal sex**‡						
Yes	6252	478 (7·6%)	0·82 (0·70–0·95)	0·0095	0·73 (0·62–0·86)	0·0001
No	3257	317 (9·7%)	1 (ref)	..	1 (ref)	..
**Condomless anal sex with partner or partners living with HIV**‡						
Yes	1892	100 (5·3%)	0·72 (0·56–0·92)	0·0097	0·80 (0·62–1·03)	0·084
No	2640	221 (8·4%)	1 (ref)	..	1 (ref)	..
I don't know	4976	474 (9·5%)	1·16 (0·98–1·37)	0·094	1·34 (1·12–1·61)	0·0011
**Transactional sex**‡						
Yes	1643	207 (12·6%)	1·51 (1·27–1·79)	<0·0001	1·35 (1·09–1·66)	0·0050
No	7865	588 (7·5%)	1 (ref)	..	1 (ref)	..
**Binge drinking**‡						
Yes	6254	556 (8·9%)	1·13 (0·96–1·32)	0·14	NA	NA
No	3254	239 (7·3%)	1 (ref)	..	NA	NA
**Stimulant use**‡§						
Yes	1743	125 (7·2%)	1·03 (0·84–1·27)	0·78	NA	NA
No	7766	670 (8·6%)	1 (ref)	..	NA	NA

OR=odds ratio. aOR=adjusted odds ratio. PrEP=pre-exposure prophylaxis. NA=not applicable.

* Attending the enrolment visit and not returning to any study visit; participants were eligible to have the event early loss to follow-up if they had time to complete 30 weeks of follow-up.

† For Brazil and Mexico: past 3 months; for Peru: past 6 months.

‡ Past 6 months.

§ Stimulant use was defined as use of any club drugs (eg, ecstasy, lysergic acid diethylamide, or gamma-hydroxybutyrate), cocaine (powder, crack, or paste), poppers, or other inhalants.

Among all enrolled participants, 6477 (68·1%) were adherent to PrEP (MPR ≥0·6), and the proportion of participants adherent was greater in Brazil than in Mexico or Peru (table 4). The odds of adherence to PrEP were lower among transgender women versus cisgender men, lower among participants aged 18–24 years and 25–30 years versus those older than 30 years, lower among participants with primary or secondary education versus those with more than secondary education, lower among participants reporting taking part in condomless anal sex with a partner or partners with unknown HIV status versus those reporting not taking part in condomless anal sex with a partner or partners living with HIV, and lower among participants reporting taking part in transactional sex versus those reporting not taking part in this behaviour (table 4). The odds of adherence to PrEP were greater among participants who attended the service seeking PrEP versus those attending for other reasons, greater among participants reporting five to ten or more than ten sex partners versus those reporting fewer than five sex partners, greater among participants reporting receptive condomless anal sex versus those not reporting this behaviour, and greater among participants reporting condomless anal sex with a partner or partners living with HIV versus those reporting not taking part in this behaviour (table 4). Similar results were observed for models per country, with some exceptions such as gender for Peru and race for Peru and Mexico (appendix 3 p 7).

**Table 4:** Factors associated with PrEP adherence measured by MPR

	**Number of participants (n=9509)**	MPR ≥0·6*(n=6477 [68·1%])	**Univariable analyses**		**Multivariable analysis**	
			OR (95% CI)	p value	aOR (95% CI)	p value
**Country**						
Brazil	3928	3053 (77·7%)	1 (ref)	..	1 (ref)	..
Mexico	3288	2315 (70·4%)	0·68 (0·61–0·76)	<0·0001	0·61 (0·54–0·69)	<0·0001
Peru	2293	1109 (48·4%)	0·27 (0·24–0·30)	<0·0001	0·38 (0·34–0·44)	<0·0001
**Gender**						
Cisgender man	8966	6222 (69·4%)	1 (ref)	..	1 (ref)	..
Transgender woman	543	255 (47·0%)	0·47 (0·39–0·56)	<0·0001	0·56 (0·46–0·70)	<0·0001
**Age, years**						
18–24	2481	1449 (58·4%)	0·49 (0·44–0·55)	<0·0001	0·52 (0·46–0·58)	<0·0001
25–30	3067	2040 (66·5%)	0·66 (0·59–0·73)	<0·0001	0·64 (0·57–0·71)	<0·0001
>30	3961	2988 (75·4%)	1 (ref)	..	1 (ref)	..
**Race**						
White	2554	1941 (76·0%)	1·21 (1·08–1·36)	0·0010	1·11 (0·99–1·25)	0·067
Black, mixed race, Indigenous, or Asian	6955	4536 (65·2%)	1 (ref)	..	1 (ref)	..
**Education**						
Primary, complete or incomplete	113	57 (50·4%)	0·42 (0·29–0·63)	<0·0001	0·60 (0·40–0·91)	0·015
Secondary, complete or incomplete	1662	915 (55·0%)	0·56 (0·50–0·63)	<0·0001	0·70 (0·61–0·79)	<0·0001
More than secondary	7734	5505 (71·2%)	1 (ref)	..	1 (ref)	..
**Main reason to attend the service**						
Seeking PrEP	8348	5938 (71·1%)	1·70 (1·48–1·95)	<0·0001	1·56 (1·35–1·80)	<0·0001
Other	1161	539 (46·4%)	1 (ref)	..	1 (ref)	..
**Number of sex partners**†						
<5	4011	2602 (64·9%)	1 (ref)	..	1 (ref)	..
5–10	2683	1866 (69·6%)	1·22 (1·09–1·36)	0·0003	1·24 (1·11–1·39)	0·0002
>10	2815	2009 (71·4%)	1·26 (1·13–1·41)	<0·0001	1·53 (1·35–1·73)	<0·0001
**Receptive condomless anal sex**‡						
Yes	6252	4345 (69·5%)	1·13 (1·03–1·24)	0·0086	1·24 (1·12–1·36)	<0·0001
No	3257	2132 (65·5%)	1 (ref)	..	1 (ref)	..
**Condomless anal sex with partner or partners living with HIV**‡						
Yes	1892	1432 (75·7%)	1·36 (1·18–1·56)	<0·0001	1·25 (1·08–1·43)	0·0021
No	2640	1756 (66·5%)	1 (ref)	..	1 (ref)	..
I don't know	4976	3288 (66·1%)	0·98 (0·88–1·08)	0·64	0·87 (0·78–0·97)	0·013
**Transactional sex**‡						
Yes	1643	960 (58·4%)	0·68 (0·70–0·76)	<0·0001	0·80 (0·69–0·91)	0·0009
No	7865	5516 (70·1%)	1 (ref)	..	1 (ref)	..
**Binge drinking**‡						
Yes	6254	4177 (66·8%)	0·90 (0·82–0·99)	0·035	0·93 (0·84–1·02)	0·15
No	3254	2299 (70·6%)	1 (ref)	..	1 (ref)	..
**Stimulant use**‡§						
Yes	1743	1224 (70·2%)	0·91 (0·81–1·03)	0·13	NA	NA
No	7766	5253 (67·6%)	1 (ref)	..	NA	NA

PrEP=pre-exposure prophylaxis. MPR=medication possession ratio. OR=odds ratio. aOR=adjusted odds ratio. NA=not applicable.

* MPR ≥0·6 is equivalent to four PrEP pills per week.

† For Brazil and Mexico: last 3 months; for Peru: last 6 months.

‡ Past 6 months.

§ Stimulant use was defined as use of any club drugs (eg, ecstasy, lysergic acid diethylamide, or gamma-hydroxybutyrate), cocaine (powder, crack, or paste), poppers, or other inhalants.

From the initial pool of enrolled participants, 1230 (12·9%) were not eligible for the long-term PrEP engagement outcome due to administrative censoring occurring before 52 weeks of follow-up. Of the 8279 eligible participants, 7556 (91·3%) attended at least one follow-up visit, while 2910 (35·1%) completed all five follow-up visits. We further excluded 54 participants who did not have the chance to complete a third visit due to adverse events (n=18), medical concerns (n=6), and HIV seroconversion (n=30). Therefore, the sample for this analysis was 8225 participants, with 5783 (70·3%) having long-term PrEP engagement (table 5). The proportion of participants with long-term PrEP engagement was greater in Brazil than in Mexico or Peru (table 5). The odds of long-term PrEP engagement were lower among transgender women versus cisgender men, lower among participants aged 18–24 years and 25–30 years versus participants older than 30 years, lower among participants with secondary education versus those with more than secondary education, and lower among participants reporting condomless anal sex with a partner or partners with unknown HIV status versus those reporting not taking part in condomless anal sex with a partner or partners living with HIV (table 5). Participants who self-reported as PrEP adherent at week 4 had three times greater odds of having long-term PrEP engagement than those who were not PrEP adherent at week 4 (table 5). The odds of long-term PrEP engagement were greater among participants who attended the service seeking PrEP versus those who attended for other reasons, greater among those reporting five to ten or more than ten sex partners versus those reporting fewer than five, greater among those reporting receptive condomless anal sex versus those reporting not taking part in this behaviour, and greater among those reporting condomless anal sex with a partner or partners living with HIV versus those reporting not taking part in this behaviour (table 5). Similar results were observed for models per country, with some exceptions such as gender for Peru and race for Peru and Mexico (appendix 3 p 9).

**Table 5:** Factors associated with long-term PrEP engagement

	**Number of participants (n=8225)**	Long-term PrEP engagement*(n=5783 [70·3%])	**Univariable analyses**		**Multivariable analysis**	
			OR (95% CI)	p value	aOR (95% CI)	p value
**Country**						
Brazil	3854	3124 (81·1%)	1 (ref)	..	1 (ref)	..
Mexico	2434	1655 (68·0%)	0·50 (0·44–0·56)	<0·0001	0·44 (0·38–0·51)	<0·0001
Peru	1937	1104 (51·8%)	0·25 (0·22–0·28)	<0·0001	0·47 (0·40–0·54)	<0·0001
**Gender**						
Cisgender man	7737	5546 (71·7%)	1 (ref)	..	1 (ref)	..
Transgender woman	488	237 (48·6%)	0·44 (0·36–0·53)	<0·0001	0·56 (0·45–0·71)	<0·0001
**Age, years**						
18–24	2208	1343 (60·8%)	0·48 (0·43–0·54)	<0·0001	0·56 (0·49–0·64)	<0·0001
25–30	2593	1799 (69·4%)	0·67 (0·60–0·76)	<0·0001	0·70 (0·61–0·79)	<0·0001
>30	3424	2641 (77·1%)	1 (ref)	..	1 (ref)	..
**Race**						
White	2391	1892 (79·1%)	1·26 (1·11–1·43)	0·0002	1·10 (0·96–1·25)	0·16
Black, mixed race, Indigenous, or Asian	5834	3891 (66·7%)	1 (ref)	..	1 (ref)	..
**Education**						
Primary, complete or incomplete	106	56 (52·8%)	0·41 (0·27–0·61)	<0·0001	0·66 (0·42–1·02)	0·063
Secondary, complete or incomplete	1515	887 (58·6%)	0·55 (0·48–0·62)	<0·0001	0·74 (0·68–0·86)	<0·0001
More than secondary	6604	4840 (73·3%)	1 (ref)	..	1 (ref)	..
**Main reason to attend the service**						
Seeking PrEP	7174	5254 (73·2%)	1·58 (1·36–1·84)	<0·0001	1·32 (1·12–1·56)	0·0007
Other	1051	529 (50·3%)	1 (ref)	..	1 (ref)	..
**Number of sex partners**†						
<5	3604	2434 (67·5%)	1 (ref)	..	1 (ref)	..
5–10	2284	1638 (71·7%)	1·25 (1·11–1·40)	0·0003	1·28 (1·12–1·45)	0·0002
>10	2337	1711 (73·2%)	1·26 (1·12–1·42)	0·0001	1·56 (1·36–1·80)	<0·0001
**Receptive condomless anal sex**‡						
Yes	5313	3805 (71·6%)	1·14 (1·03–1·26)	0·012	1·24 (1·11–1·39)	0·0001
No	2912	1978 (67·9%)	1 (ref)	..	1 (ref)	..
**Condomless anal sex with a partner or partners living with HIV**‡						
Yes	1652	1280 (77·5%)	1·34 (1·16–1·56)	0·0001	1·19 (1·01–1·39)	0·032
No	2366	1642 (69·4%)	1 (ref)	..	1 (ref)	..
I don't know	4207	2861 (68·0%)	0·92 (0·82–1·03)	0·17	0·82 (0·73–0·93)	0·0020
**Transactional sex**‡						
Yes	1324	776 (58·6%)	0·65 (0·57–0·73)	<0·0001	0·81 (0·69–0·95)	0·080
No	6901	5007 (72·6%)	1 (ref)	..	1 (ref)	..
**Binge drinking**‡						
Yes	5406	3769 (69·7%)	0·98 (0·89–1·09)	0·78	NA	NA
No	2819	2014 (71·4%)	1 (ref)	..	NA	NA
**Stimulant use**‡§						
Yes	1510	1101 (72·9%)	1·01 (0·89–1·15)	0·85	NA	NA
No	6715	4682 (69·7%)	1 (ref)	..	NA	NA
**Self-reported PrEP adherence at week 4**¶						
Yes	4521	3759 (83·2%)	3·50 (3·15–3·89)	<0·0001	3·14 (2·82–3·50)	<0·0001
No	3704	2024 (54·6%)	1 (ref)	..	1 (ref)	..

PrEP=pre-exposure prophylaxis. OR=odds ratio. aOR=adjusted odds ratio. NA=not applicable.

* Attending the week 4 study visit plus two or more quarterly visits within a 52-week follow-up period; participants were eligible to have the event long-term PrEP engagement if they had time to complete 52-weeks of follow-up.

† For Brazil and Mexico: past 3 months; for Peru: past 6 months.

‡ Past 6 months.

§ Stimulant use was defined as use of any: club drugs (eg, ecstasy, lysergic acid diethylamide, or gamma-hydroxybutyrate), cocaine (powder, crack, or paste), poppers, or other inhalants.

¶ Report of any missing pill in the previous 30 days.

Prevalence of active syphilis at enrolment was 8·8% (95% CI 8·2–9·4), and overall incidence was 10·09 per 100 person-years (95% CI 9·40–10·82; appendix 3 p 11). Syphilis incidence was greater in Brazil than in Peru or Mexico, and among transgender women than cisgender men. Prevalence of rectal chlamydia decreased from enrolment to week 52 (appendix 3 p 12). Prevalence of gonorrhoea also decreased from enrolment to week 52 (appendix 3 p 12). Among participants aged 18–24 years and transgender women, prevalence of both chlamydia and gonorrhoea at enrolment were greater than in the overall population, although they did not increase over time (appendix 3 p 12). Prevalence of both chlamydia and gonorrhoea decreased over time among participants from Mexico and Peru but remained stable among participants from Brazil (appendix 3 p 12).

Prevalence of active hepatitis B remained stable from enrolment to week 52, whereas prevalence of hepatitis C increased over time (appendix 3 p 13). Prevalence of hepatitis C increased over time among participants from Mexico and Peru, cisgender men, and those aged 25–30 years.

Overall, 87 (0·91%) of 9509 participants interrupted PrEP due to adverse events, and 52 (0·55%) discontinued PrEP due to adverse events (appendix 3 p 14). Notably, very few participants interrupted PrEP (31 [0·33%]) or discontinued PrEP (14 [0·15%]) due to creatinine clearance of 60 mL/min or less.

## Discussion

In this large cohort of 9509 MSM and transgender women enrolled in Brazil, Mexico, and Peru, a same-day PrEP delivery strategy was shown to be feasible and effective, with only a small proportion of participants with early loss to follow-up. Most participants had PrEP adherence and long-term PrEP engagement, although country, sociodemographic, and behavioural disparities were observed. Although HIV incidence was low during the study compared with Latin American sites participating in the HPTN083 study (tenofovir disoproxil fumarate plus emtricitabine group)^15^ and the placebo group in the iPrEX study for Brazil (5·0 per 100 person-years) and Peru (3·5 per 100 person-years),^16^ it was greater than that observed in implementation studies conducted in France and Australia.^3^, ^17^ Participants from Peru, transgender women, younger participants (aged 18–24 years), and individuals who were not White had greater HIV incidence, as well as increased odds of early loss to follow-up and lower odds of PrEP adherence and long-term PrEP engagement. Our results corroborate the finding that early adherence, measured at week 4, is associated with higher likelihood of long-term PrEP engagement.^18^ The first month after PrEP initiation can be a valuable window of opportunity to implement strategies aiming to improve PrEP outcomes, particularly among the most clinically vulnerable.

The moderately high prevalence (0·5%) of acute HIV infection, in agreement with that of the PrEP Brasil study^19^ but not quite as high as in the iPrEx study,^16^ suggests that MSM and transgender women interested in PrEP are highly vulnerable to HIV. The opportunity to identify individuals with acute HIV infection before PrEP initiation is particularly important with the introduction of novel long-acting PrEP technologies, such as injectable cabotegravir.^15^ Initiation of PrEP in individuals with non-diagnosed acute HIV infection might lead to HIV drug resistance, potentially affecting treatment outcomes.^20^

Our results underscore the importance of PrEP adherence to prevent new HIV infections, as shown by the lower HIV incidence among participants with an MPR of 0·6 or more. The association of younger age with higher HIV incidence is in line with trends observed in Latin America showing a higher number of new HIV cases among this subgroup.^10^ A combination of lower adherence and lower long-term PrEP engagement might explain these worse outcomes. In a recent cross-sectional online survey conducted in Brazil, younger age was associated with decreased odds of high perceived HIV risk,^21^ which can negatively affect PrEP outcomes. The higher HIV incidence among Peruvian participants might be explained by the inclusion of a younger population, more individuals enrolled who were not actively seeking PrEP, lower adherence, and lower long-term PrEP engagement. Moreover, lower PrEP awareness might explain the lower proportion of participants seeking PrEP in Peru than in Brazil and Mexico.^22^

Low early loss to follow-up indicates the feasibility of same-day PrEP prescribing and delivery, which reduces the attrition between initial evaluation and receipt of a PrEP prescription, as previously seen in the USA.^9^ Our results expand this body of evidence from the perspective of PrEP services in Latin America, and could also be useful for other resource-limited regions. Moreover, our results reinforce that same-day PrEP, as adopted by the Ministry of Health in Brazil since 2017 and in Mexico since 2021, should be maintained and serve as a model for other countries in the region.

ImPrEP participants with lower education compared with higher education in all countries, and of Black or mixed race compared with those of White race in Brazil had worse PrEP outcomes. Social determinants of health, such as race and education, contribute to widening health disparities and inequalities.^23^ Furthermore, MSM and transgender women are also exposed to recurring marginalising experiences, such as homophobia and transphobia, which might affect mental health and wellbeing.^24^ Black and less educated MSM and transgender women are additionally affected by intersectional discrimination related to racism and classism. Studies evaluating PrEP outcomes in Brazil found that Black MSM had lower PrEP awareness, PrEP uptake, and PrEP adherence than White MSM.^18^, ^22^, ^25^ PrEP services in Latin America should focus on strategies to and explore and address stigma and to encourage PrEP reinitiation, adherence, and persistence among these vulnerable populations, with interventions addressing the gap in access and retention considering age, race, and education.

Young MSM and transgender women aged 18–24 years had worse PrEP outcomes compared with older peers. In Latin America, young MSM are more vulnerable to HIV infection due to poor access to comprehensive sex education and preventive strategies, condomless and PrEP-less sex, and low perceived HIV risk.^21^, ^26^ HIV prevalence has been increasing in the past decade in the region among young MSM,^10^ who show lower PrEP awareness, willingness, uptake, and adherence compared with older MSM.^18^, ^22^, ^25^ Alternative approaches to recruit and retain young populations in PrEP services should be explored, such as using new social media platforms (eg, TikTok), engaging them in development of digital campaigns, use of appropriate language, and having digital influencers to better communicate with these groups.^27^

Transgender women had worse PrEP outcomes when compared with cisgender men, corroborating findings from previous studies.^18^, ^28^ Transgender women consistently face challenges engaging in prevention and treatment services, reflecting their underlying vulnerabilities and poor adaptation of services to their needs. A transgender-specific PrEP study in Brazil showed that although high engagement in prevention services was attainable, PrEP adherence decreased over time, with social determinants of health substantially affecting adherence during the study follow-up.^29^ High PrEP retention was achieved in settings where staff were trained on transgender competency, and where gender-affirming care with feminising hormone therapy was provided as part of the study.^29^ In a Peruvian study to support PrEP among transgender women, more than half of participants (49 [55%] of 89) were lost to follow-up after 3 months.^28^ Stigma and previous discriminatory experiences in the health-care setting are crucial barriers to HIV prevention, including PrEP, among transgender women.^30^

The country-level differences observed are likely to reflect distinct underlying characteristics of the populations enrolled, the effect of COVID-19 pandemic restrictive measures, and particularities of countries' public health systems. Brazil, Mexico, and Peru together represent more than half of the Latin American population (approximately 368 million). These countries have vast socioeconomic disparities, which intensified during the COVID-19 pandemic. Key populations were particularly affected, including by reduced access to HIV prevention services.^31^ A substantial part of the follow-up was affected by the restrictive measures of the pandemic, especially in Peru. Brazil was less affected due to available telehealth procedures, including HIV self-testing distribution for PrEP delivery.^32^

The incidence of sexually transmitted infections was greater among younger participants and transgender women. Syphilis incidence during follow-up was similar in the PrEP Brasil study,^18^ although prevalence at enrolment was lower than the 2010–20 pooled prevalence in Latin America and the Caribbean (8·8% *vs* 11·2%).^33^ High prevalence of rectal chlamydia and gonorrhoea before PrEP initiation, and decreased prevalence at week 52 suggest that risk compensation did not play a role in our study.

A major strength of this study is its sample size, making it, to our knowledge, the largest PrEP cohort in Latin America. Notably, ImPrEP included the second largest absolute number of transgender women among all PrEP clinical trials and implementation studies to date (n=543). Furthermore, the study was implemented in Brazil at public health system sites, where most of the Brazilian population access PrEP, and contributed to PrEP implementation in Mexico. Some limitations should be acknowledged. We could not perform counterfactual calculations because there was no comparator group or recent incidence data in the region. Our results are not informative of PrEP uptake because participants who were screened and enrolled had previously expressed an interest in participating in the study. Data on PrEP refusal and qualitative data on individuals who had early loss to follow-up or low long-term PrEP engagement or adherence were not collected. We used different timeframes to evaluate number of sex partners across countries. PrEP adherence data were based on self-report and MPR and not on biomarkers of adherence. Although self-reported adherence could overestimate adherence, be context-dependent, and be limited by different biases, previous analyses have shown that MPR and self-reported adherence can discriminate against participants with and without protective tenofovir diphosphate levels in Brazil.^13^ Study person-years, although longer than in most studies, is still short when compared with a person's lifetime experience of HIV prevention. Studies with longer follow-up will be required to address the sustainability of long-term daily PrEP more comprehensively, and to measure the complexity of how MSM and transgender women start and stop PrEP in relation to HIV vulnerability.

In conclusion, same-day oral PrEP implementation among MSM and transgender women was feasible in Latin America, with low early loss to follow-up. Although PrEP adherence and long-term PrEP engagement were high, disparities were observed among the populations most vulnerable to HIV infection, which might jeopardise the overall efficacy of PrEP programmes. Despite PrEP use, HIV incidence remained high among transgender women, younger and non-White individuals, and participants who were not adherent to PrEP. Long-acting PrEP strategies might overcome these identified pitfalls.

## Data sharing

A complete de-identified dataset sufficient to reproduce the primary study findings will be made available on request to the corresponding author, following approval of a concept sheet summarising the analyses to be done.

## Declaration of interests

JVM declares support from Pfizer, Janssen Pharmaceutica, ViiV Healthcare, Gilead, and Merck Sharp and Dohme; being a member of an advisory board for Gilead, ViiV, and Janssen Pharmaceutica; and coordination of the AIDS Committee of the Brazilian Infectology Society. All other authors declare no competing interests.

## Supplementary Material

For Portuguese Summary translation

For Spanish Summary translation
