# Supplementary material for: Same-day initiation of oral pre-exposure prophylaxis among gay, bisexual, and other cisgender men who have sex with men and transgender women in Brazil, Mexico, and Peru (ImPrEP): a prospective, single-arm, open-label, multicentre implementation study
Source: Lancet HIV. 2022 Dec 21;10(2):e84–96. doi: 10.1016/S2352-3018(22)00331-9 (PMC9889521; doi:10.1016/S2352-3018(22)00331-9)
Supplement: For Portuguese Summary translation [file mmc1.pdf]

THE LANCET
HIV

Supplementary appendix 1

This translation in Portuguese was submitted by the authors and we reproduce it as supplied. It has not been peer reviewed. The Lancet's editorial processes have only been applied to the original in English, which should serve as reference for this manuscript.

Esta tradução em português foi submetida pelos autores e nós não fizemos quaisquer alterações. Esta versão não foi revista por pares. O processo editorial do The Lancet só foi aplicado à versão original em inglês, que deve servir como referência para este artigo.

Supplement to: Veloso VG, Cáceres CF, Hoagland B, et al. Same-day initiation of oral pre-exposure prophylaxis among gay, bisexual, and other cisgender men who have sex with men and transgender women in Brazil, Mexico, and Peru (ImPrEP): a prospective, single-arm, open-label, multicentre implementation study. *Lancet HIV* 2022; published online Dec 21. https://doi.org/10.1016/S2352-3018(22)00331-9.

Resumo em português

Introdução: Embora gays, bissexuais e outros homens cisgênero que fazem sexo com homens (HSH), travestis e mulheres trans sejam as populações mais afetadas pelo HIV na América Latina, a implementação da profilaxia pré-exposição (PrEP) é limitada. O estudo de Implementação da PrEP (ImPrEP) teve como objetivo avaliar a viabilidade da oferta de início imediato de PrEP oral no Brasil, Peru e México.

Métodos: O ImPrEP foi um estudo de implementação prospectivo, de braço único, aberto e multicêntrico conduzido no Brasil (14 centros), México (quatro centros), e Peru (10 centros). HSH e travestis e mulheres trans foram elegíveis para participar se tivessem 18 anos ou mais, teste de HIV negativo, e relatassem um ou mais critérios pré-especificados. Participantes incluídos receberam no mesmo dia PrEP oral para uso diário (fumarato de tenofovir desoproxila [300mg] coformulado com entricitabina [200mg]). As visitas de acompanhamento foram agendadas para a semana 4 e, posteriormente, trimestralmente. Modelos de regressão logística foram usados para identificar fatores associados à perda precoce de acompanhamento (não retornar ao serviço após a inclusão no estudo), adesão à PrEP (taxa de posse de medicamento ≥0,6) e retenção a longo prazo (comparecimento a 3 ou mais visitas em 52 semanas). O estudo foi registrado na Base de Ensaios Clínicos do Brasil (ReBEC), U1111-1217-6021.

Resultados: De 6 de fevereiro de 2018 a 30 de junho de 2021, 9.979 participantes foram triados e 9.509 foram incluídos no estudo (Brasil n=3.928, México n=3.288, Peru n=2.293). 543 (5,7%) participantes eram travestis e mulheres trans, 8.966 (94,3%) homens cis, e 2.481 (26,1%) tinham idade entre 18 e 24 anos, com um total de 12.185,25 pessoas-ano de acompanhamento. A incidência de HIV foi de 0,85/100 pessoas-ano (IC 95%: 0,70-1,03), e foi maior em travestis e mulheres trans, participantes do Peru, indivíduos de 18 a 24 anos, negros e com baixa adesão à PrEP. Um total de 795 entre 9.509 (8,4%) participantes tiveram perda precoce de acompanhamento, 6.477 (68,1%) de 9.509 aderiram à PrEP e 5.783 (70,3%) entre 8.225 tiveram retenção a longo prazo. Travestis e mulheres trans (razões de chance ajustada 1,60; IC 95% 1,20-2,14]), participantes com 18-24 anos (1,80;

1,49-2,18]) e aqueles apenas com ensino fundamental (2,18; 1,29-3,68]) tiveram chances aumentadas de perda precoce de acompanhamento. Travestis e mulheres trans (0,56; 0,46-0,70), participantes com 18-24 anos (0,52; 0,46-0,58) e aqueles com ensino fundamental (0,60; 0,40-0,91) tiveram menores chances de adesão à PrEP. Travestis e mulheres trans (0,56; 0,45-0,71), participantes com 18-24 anos (0,56; 0,49-0,64) e aqueles com ensino médio (0,74; 0,68-0,86) tiveram menores chances de retenção a longo prazo. A incidência de sífilis foi de 10,09/100 pessoas-ano (IC 95%: 9,40-10,82). A prevalência de infecção retal por Clamídia e gonorreia na inclusão foram 9,2% (IC 95%: 8,6-9,8) e 11,8% (IC 95%: 11,8-12,4), respectivamente, e diminuíram ao longo do estudo.

Interpretação: O início imediato de PrEP oral diária é uma estratégia viável para HSH, travestis e mulheres trans na América Latina. Os determinantes sociais e estruturais de vulnerabilidade para o HIV precisam ser considerados e abordados para a plena realização dos benefícios da PrEP.
