# Supplementary material for: Same-day initiation of oral pre-exposure prophylaxis among gay, bisexual, and other cisgender men who have sex with men and transgender women in Brazil, Mexico, and Peru (ImPrEP): a prospective, single-arm, open-label, multicentre implementation study
Source: Lancet HIV. 2022 Dec 21;10(2):e84–96. doi: 10.1016/S2352-3018(22)00331-9 (PMC9889521; doi:10.1016/S2352-3018(22)00331-9)
Supplement: For Spanish Summary translation [file mmc2.pdf]

THE LANCET
HIV

Supplementary appendix 2

This translation in Spanish was submitted by the authors and we reproduce it as supplied. It has not been peer reviewed. *The Lancet*'s editorial processes have only been applied to the original in English, which should serve as reference for this manuscript.

Los autores nos proporcionaron esta traducción al español y la reproducimos tal como nos fue entregada. No la hemos revisado. Los procesos editoriales de *The Lancet* se han aplicado únicamente al original en inglés, que debe servir de referencia para este manuscrito.

Supplement to: Veloso VG, Cáceres CF, Hoagland B, et al. Same-day initiation of oral pre-exposure prophylaxis among gay, bisexual, and other cisgender men who have sex with men and transgender women in Brazil, Mexico, and Peru (ImPrEP): a prospective, single-arm, open-label, multicentre implementation study. *Lancet HIV* 2022; published online Dec 21. https://doi.org/10.1016/S2352-3018(22)00331-9.

Resumen en español

Antecedentes: Aunque los hombres cisgénero homosexuales, bisexuales y otros que tienen sexo con hombres (HSH) y las mujeres transgénero (MT) sufren la mayor carga de VIH en Latinoamérica, la implementación de la profilaxis preexposición (PrEP) es notablemente limitada. El estudio de Implementation PrEP (ImPrEP) tuvo como objetivo evaluar la viabilidad de la dispensación inmediata de la PrEP oral en Brasil, Perú y México.

Métodos: El ImPrEP fue un estudio de implementación prospectivo, de un solo brazo, abierto y multicéntrico desarrollado en Brasil (14 centros), México (quatro centros) y Perú (10 centros). Los HSH y MT fueron elegibles si tenían 18 años o más, prueba negativa para VIH y uno o más criterios preseleccionados. Los participantes recibieron el mismo día de la inclusión la PrEP oral para tomar diariamente (fumarato de tenofovir disoproxil [300mg] coformulado con emtricitabina [200mg]). Las visitas de seguimiento se programaron a la cuarta semana y después trimestralmente. Se utilizaron modelos de regresión logística para identificar los factores asociados con la pérdida temprana al seguimiento (NRDI; no regreso después de la inclusión), la adherencia a la PrEP (tasa de posesión del medicamento ≥0.6) y la retención a largo plazo (asistencia a 3+ visitas en 52 semanas). Núm. Id. del estudio en la Base de Ensayos Clínicos de Brasil (ReBEC): U1111-1217-6021.

Resultados: Del 6-febrero-2018 al 30-junio-2021, 9,979 participantes fueron tamizados y se incluyeron 9,509 participantes (Brasil n=3,928, México n=3,288, Perú n=2,293). De estos, 543 (5.7%) participantes eran MT, 8,966 (94.3%) hombres cis y 2,481 (26.1%) de 18-24 años. Hubo 12,185.25 años-persona de seguimiento. La incidencia del VIH fue de 0.85/100 años-persona (I.C. 95%: 0.70-1.03), y fue mayor en MT, participantes de Perú, los de 18-24 años, negros/mestizos y no adherentes a la PrEP. Un total de 795 de 9,509 (8.4%) participantes tuvieron NRDI; 6,477 de 9,509 (68.1%) fueron adherentes a la PrEP y 5,783 de 8,225 (70.3%) tuvieron retención a largo plazo. Las MT (razón de momios ajustada [RMa]: 1.60; I.C. 95%:1.20-2.14), los participantes de 18-24 años (RMa: 1.80; 1.49-2.18) y aquellos con educación primaria (RMa: 2.18; 1.29-3.68) tuvieron mayor razón de momios de NRDI. Las MT (RMa: 0.56; 0.46-0.70), los participantes de 18-24 años (RMa: 0.52;

0.46-0.58) y aquellos con educación secundaria (RMa: 0.70; 0.61-0.79) tuvieron menor razón de momios de adherencia a la PrEP. Las MT (RMa: 0.56; 0.45-0.71), los participantes de 18-24 años (RMa: 0.56; 0.49-0.64) y aquellos con educación primaria (RMa: (0,60; 0,40-0,91) tuvieron menor razón de momios de retención a largo plazo. La incidencia de sífilis fue de 10.09/100 años-persona (I.C. 95%:9.40-10.82). La prevalencia de clamidia y gonorrea rectal durante la inclusión fue de 9.2% (I.C. 95%:8.6-9.8) y 11.8% (I.C. 95%:11.8-12.4), respectivamente, pero disminuyeron a lo largo del tiempo.

Interpretación: La dispensación inmediata de la PrEP es viable para los HSH y MT de Latinoamérica. Los determinantes sociales y estructurales del riesgo al VIH necesitan ser abordados para alcanzar plenamente los beneficios de la PrEP.
