# Supplementary material for: Same-day initiation of oral pre-exposure prophylaxis among gay, bisexual, and other cisgender men who have sex with men and transgender women in Brazil, Mexico, and Peru (ImPrEP): a prospective, single-arm, open-label, multicentre implementation study
Source: Lancet HIV. 2022 Dec 21;10(2):e84–96. doi: 10.1016/S2352-3018(22)00331-9 (PMC9889521; doi:10.1016/S2352-3018(22)00331-9)
Supplement: Supplementary appendix 3 [file mmc3.pdf]

THE LANCET
HIV

Supplementary appendix 3

This appendix formed part of the original submission and has been peer reviewed. We post it as supplied by the authors.

Supplement to: Veloso VG, Cáceres CF, Hoagland B, et al. Same-day initiation of oral pre-exposure prophylaxis among gay, bisexual, and other cisgender men who have sex with men and transgender women in Brazil, Mexico, and Peru (ImPrEP): a prospective, single-arm, open-label, multicentre implementation study. *Lancet HIV* 2022; published online Dec 21. https://doi.org/10.1016/S2352-3018(22)00331-9.

Supplementary Material

	Page
Supplementary Figure 1: Study flowchart.	2
Supplementary Table 1: Participants reenrolled during the ImPrEP study.	3
Supplementary Table 2: Factors associated with early loss to follow-up (ELFU) per country.	5
Supplementary Table 3: Factors associated with PrEP adherence measured by medication possession ratio (MPR) according to country.	7
Supplementary Table 4: Factors associated with long-term PrEP engagement according to country.	9
Supplementary Table 5: Prevalence of syphilis at enrollment and syphilis incidence during study follow-up overall and stratified per country, age, and gender.	11
Supplementary Table 6: Proportion of participants with rectal chlamydia and gonorrhea at enrollment and week 52 overall and stratified per country, gender, and age.	12
Supplementary Table 7: Proportion of participants with hepatitis B and hepatitis C at enrollment and week 52 overall and stratified per country, gender, and age.	13
Supplementary Table 8: Number of individuals that interrupted and discontinued PrEP due to adverse events.	14
ImPrEP Study Group	15
ImPrEP Study Sites	16

Supplementary Figure 1· Study flowchart.

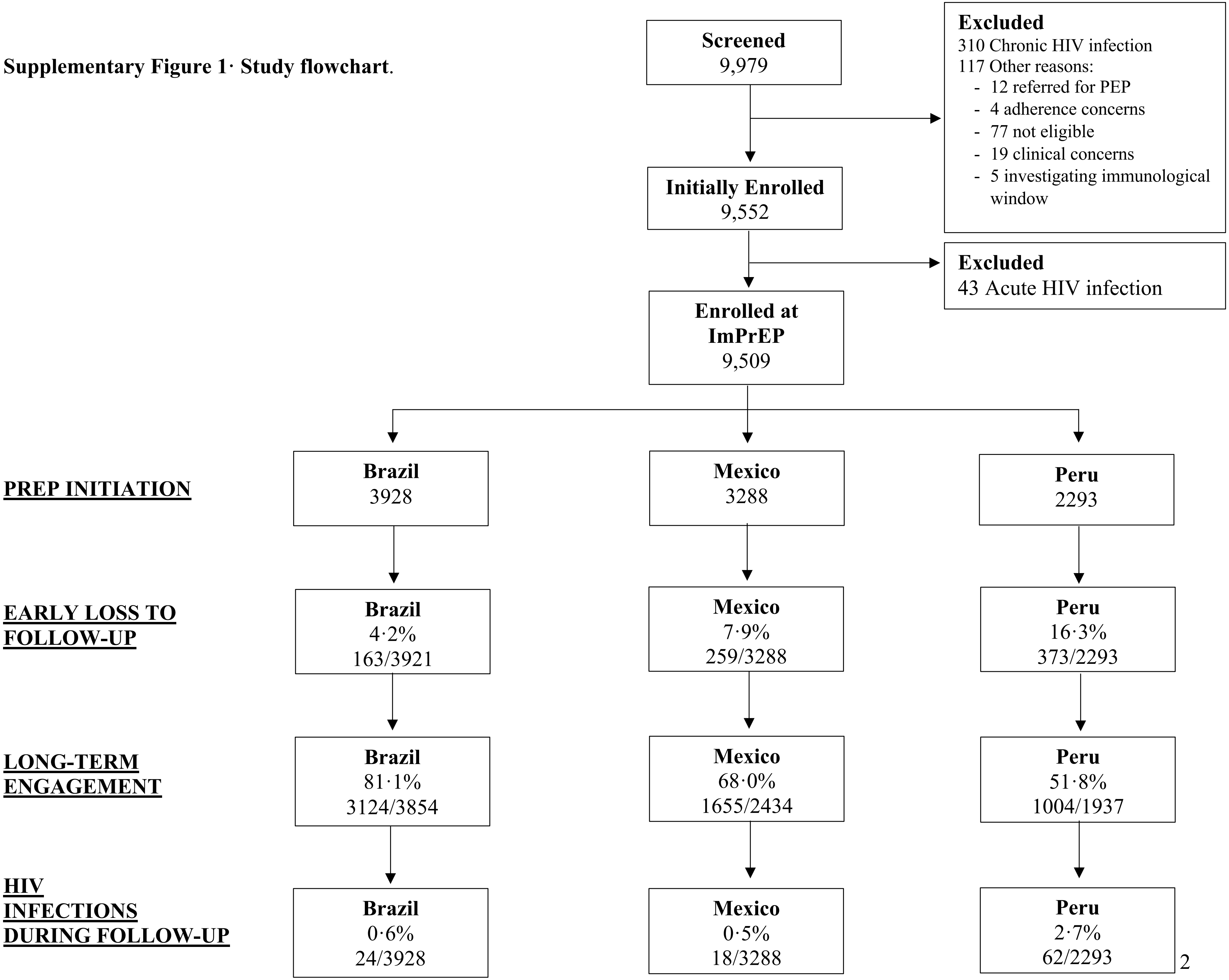

ELFU was defined as attending the enrollment visit and not returning to any follow-up visit. Long-term PrEP engagement was defined as attending the week 4 study visit plus two or more quarterly visits within a 52-week follow-up period.

Supplementary Table 1. Participants reenrolled during the ImPrEP study.

Number of enrollments	Overall		Brazil		Mexico		Peru	
	**N**	**%**	**N**	**%**	**N**	**%**	**N**	**%**
1	8594	90·4	3696	94·1	3223	98·0	1675	73·1
2	850	8·9	216	5·5	65	2·0	569	24·8
3	59	0·6	12	0·3	--	--	47	2·0
4	6	0·1	4	0·1	--	--	2	0·1
**Total**	**9509**	**100·0**	**3928**	**100·0**	**3288**	**100·0**	**2293**	**100·0**

Supplementary Table 2. Factors associated with early loss to follow-up (ELFU) per country.

	Brazil				Mexico				Peru			
	N	ELFU[1] (%)	**Univariable analyses** OR (95%CI)	**Multivariable analysis** aOR (95%CI)	N	ELFU[1] (%)	**Univariable analyses** OR (95%CI)	**Multivariable analysis** aOR (95%CI)	N	ELFU[1] (%)	**Univariable analyses** OR (95%CI)	**Multivariable analysis** aOR (95%CI)
**Gender**												
Cisgender man	3733	3·6	1	1	3193	7·5	1	1	2040	16·0	1	NA
Transgender woman	195	13·8	4·25 (2·73-6·60)	**2·82 (1·58-5·03)**	95	21·0	3·30 (1·98-5·49)	**2·38 (1·34-4·23)**	253	18·2	1·16 (0·83-1·64)	NA
**Age (years)**												
18-24	1033	6·2	2·78 (1·85-4·16)	**2·19 (1·44-3·34)**	643	12·0	2·09 (1·52-2·87)	**1·75 (1·25-2·43)**	805	19·0	1·52 (1·16-1·99)	**1·57 (1·19-2·08)**
25-30	1216	4·9	2·18 (1·45-3·29)	**2·05 (1·35-3·10)**	1156	7·9	1·31 (0·97-1·77)	1·25 (0·92-1·70)	695	16·4	1·27 (1·95-1·69)	**1·50 (1·11-2·01)**
>30	1679	2·3	1	1	1489	6·1	1	1	793	13·4	1	1
**Race**												
Black/Mix-race/Indigenous/Asian	2060	5·3	1	1	2835	8·2	1	1	2060	16·2	1	NA
White	1868	2·9	0·53 (0·38-0·74)	**0·66 (0·47-0·64)**	453	5·5	0·65 (0·42-0·99)	0·71 (0·46-1·10)	233	17·2	1·07 (0·75-1·54)	NA
**Education**												
Primary (complete or incomplete)	52	9·6	3·22 (1·26-8·29)	1·57 (0·56-4·42)	23	17·4	2·70 (0·91-8·00)	1·72 (0·56-5·35)	38	31·6	3·06 (1·52-6·16)	**2·91 (1·42-5·97)**
Secondary (complete or incomplete)	775	7·6	2·50 (1·79-3·48)	**1·68 (1·16-2·42)**	182	17·6	2·74 (1·82-4·10)	**1·87 (1·20-2·92)**	705	22·4	1·92 (1·52-2·41)	**1·85 (1·46-2·35)**
More than secondary	3101	3·2	1	1	3083	7·2	1	1	1550	13·1	1	1
**Main reason to attend the service**												
Seeking PrEP	3774	4·1	0·78 (0·38-1·62)	NA	3126	7·8	0·83 (0·48-1·43)	NA	1448	12·8	0·52 (0·41-0·65)	**0·56 (0·44-0·70)**
Other	154	5·2	1	NA	162	9·3	1	NA	845	22·1	1	1
**Number of sex partners[2]**												
< 5	1813	4·8	1	1	1045	9·7	1	1	1153	18·3	1	1
5-10	968	3·3	0·67 (0·44-1·01)	**0·66 (0·43-1·02)**	1107	8·2	0·84 (0·62-1·13)	0·78 (0·57-1·06)	608	14·6	0·76 (0·58-1·00)	0·85 (0·64-1·12)
> 10	1147	3·8	0·76 (0·53-1·11)	**0·41 (0·26-0·66)**	1136	5·9	0·58 (0·42-0·81)	**0·46 (0·32-0·66)**	532	13·7	0·71 (0·53-0·95)	**0·68 (0·51-0·92)**
**Receptive CAS[2]**												
Yes	2557	4·1	0·97 (0·70-1·34)	NA	2340	7·3	0·76 (0·58-0·99)	**0·74 (0·56-0·98)**	1355	15·0	0·80 (0·64-1·00)	**0·74 (0·59-0·93)**
No	1371	4·2	1	NA	948	9·4	1	1	938	18·1	1	1
**CAS with partner(s) living with HIV[3]**												
Yes	824	3·4	1·00 (0·61-1·63)	1·01 (0·61-1·67)	831	5·4	0·60 (0·41-0·90)	0·70 (0·47-1·04)	237	11·4	0·69 (0·44-1·08)	NA
No	1144	3·4	1	1	752	8·6	1	1	744	15·7	1	NA
I don't know	1960	4·9	1·46 (1·00-2·13)	**1·54 (1·04-2·28)**	1704	8·7	1·01 (0·75-1·37)	1·17 (0·86-1·61)	1312	17·4	1·13 (0·89-1·44)	NA
**Transactional sex[3]**												
Yes	397	8·8	2·57 (1·74-3·79)	**1·79 (1·02-3·13)**	732	10·4	1·50 (1·13-1·99)	**1·40 (1·00-1·95)**	514	18·7	1·24 (0·96-1·61)	NA
No	3531	3·6	1	1	2555	7·2	1	1	1779	15·6	1	NA
**Binge drinking[3]**												

Yes	2566	4·0	0·93 (0·67-1·29)	NA	2009	8·4	1·19 (0·91-1·55)	NA	1679	16·9	1·20 (0·93-1·56)	NA
No	1362	4·3	1	NA	1278	7·1	1	NA	614	14·5	1	NA
**Stimulant use[3,4]**												
Yes	715	4·6	1·15 (0·78-1·70)	NA	888	7·6	0·94 (0·70-1·25)	NA	140	17·9	1·13 (0·72-1·76)	NA
No	3213	4·0	1	NA	2400	8·0	1	NA	2153	16·2	1	NA

Bold: p≥0·05; OR: Odds Ratio, aOR: adjusted OR, 95%CI: 95% Confidence Interval, NA: Not Applicable. [1]Attending the enrollment visit and not returning to any study visit. Participants were eligible to have the event ELFU if they had time to complete 30 weeks of follow-up; [2]For Brazil and Mexico: last 3 months, for Peru: last 6 months; [3]last 6 months; [4]stimulant use was defined as use of any: club drugs (e.g. ecstasy, LSD and GHB), cocaine (powder, crack, or paste), poppers or other inhalants.

Supplementary Table 3. Factors associated with PrEP adherence measured by medication possession ratio (MPR) according to country.

	Brazil				Mexico				Peru			
	N	MPR ≥ 0·60	Univariable analyses	Multivariable analysis	N	MPR ≥ 0·60	Univariable analyses	Multivariable analysis	N	MPR ≥ 0·60	Univariable analyses	Multivariable analysis
			OR (95%CI)	aOR (95%CI)			OR (95%CI)	aOR (95%CI)			OR (95%CI)	aOR (95%CI)
**Gender**												
Cisgender man	3733	78·7	1	1	3193	71·2	1	1	2040	49·5	1	1
Transgender woman	195	58·5	0·38 (0·28-0·51)	**0·51 (0·36-0·74)**	95	44·2	0·32 (0·21-0·48)	**0·36 (0·23-0·56)**	253	39·1	0·66 (0·50-0·86)	1·02 (0·75-1·40)
**Age (years)**												
18-24	1033	68·3	0·38 (0·31-0·45)	**0·42 (0·35-0·51)**	643	61·7	0·51 (0·42-0·62)	**0·55 (0·45-0·68)**	805	43·0	0·65 (0·54-0·80)	**0·64 (0·52-0·78)**
25-30	1216	75·4	0·70 (0·58-0·85)	**0·55 (0·46-0·67)**	1156	67·9	0·76 (0·62-0·93)	**0·68 (0·57-0·81)**	695	48·6	0·80 (0·65-0·98)	0·92 (0·74-1·13)
>30	1679	85·2	1	1	1489	76·1	1	1	793	53·6	1	1
**Race**												
Black/Mix-race/Indigenous/Asian	2060	74·5	1	1	2835	70·4	1	NA	2070	48·8	1	NA
White	1868	81·3	1·48 (1·27-1·73)	**1·28 (1·10-1·50)**	453	70·6	1·01 (0·81-1·26)	NA	233	44·2	0·83 (0·63-1·09)	NA
**Education**												
Primary (complete or incomplete)	52	57·7	0·34 (0·20-0·60)	**0·52 (0·28-0·94)**	23	60·9	0·63 (0·27-1·46)	0·93 (0·39-2·24)	38	34·2	0·45 (0·23-0·89)	0·58 (0·28-1·17)
Secondary (complete or incomplete)	775	70·2	0·59 (0·49-0·70)	**0·81 (0·67-0·98)**	182	57·7	0·55 (0·41-0·75)	0·75 (0·54-1·04)	705	37·7	0·52 (0·44-0·63)	**0·61 (0·50-0·74)**
More than secondary	3101	79·9	1	1	3083	71·2	1	1	1550	53·6	1	1
**Main reason to attend the service**												
Seeking PrEP	3774	78·0	1·46 (1·03-2·09)	1·39 (0·96-2·00)	3126	70·4	1·03 (0·73-1·46)	NA	1448	54·7	2·01 (1·69-2·39)	**1·82 (1·52-2·19)**
Other	154	70·8	1	1	162	70·0	1	NA	845	37·5	1	1
**Number of sex partners**[3]												
< 5	1813	75·9	1	1	1045	65·8	1	1	1153	46·7	1	NA
5-10	968	81·5	1·40 (1·15-1·70)	**1·28 (1·05-1·56)**	1107	69·9	1·21 (1·01-1·44)	**1·25 (1·04-1·51)**	608	49·8	1·14 (0·93-1·38)	NA
> 10	1147	77·4	1·09 (0·91-1·30)	**1·30 (1·06-1·58)**	1136	75·1	1·56 (1·30-1·88)	**1·77 (1·44-2·17)**	532	50·4	1·16 (0·94-1·42)	NA
**Receptive CAS**[4]												
Yes	2557	78·4	1·13 (0·96-1·32)	NA	2340	71·6	1·21 (1·03-1·43)	**1·22 (1·03-1·44)**	1355	49·0	1·06 (0·90-1·26)	NA
No	1371	76·4	1	NA	948	67·5	1	1	938	47·4	1	NA
**CAS with partner(s) living with HIV**[4]												
Yes	824	80·2	1·10 (0·88-1·38)	NA	831	75·1	1·41 (1·13-1·76)	**1·27 (1·02-1·60)**	237	62·0	1·89 (1·40-2·55)	**1·67 (1·22-2·27)**
No	1144	78·6	1	NA	752	68·1	1	1	744	46·4	1	1
I don't know	1960	76·2	0·87 (0·73-1·04)	NA	1704	69·1	1·05 (0·87-1·26)	0·92 (0·76-1·12)	1312	47·0	1·03 (0·86-1·23)	1·01 (0·84-1·22)
**Transactional work**[4]												
Yes	397	66·5	0·53 (0·42-0·66)	**0·74 (0·56-0·99)**	732	66·5	0·79 (0·66-0·94)	0·84 (0·69-1·04)	514	40·7	0·67 (0·55-0·82)	0·88 (0·71-1·11)
No	3531	79·0	1	1	2555	71·5	1	1	1779	50·6	1	1
**Binge drinking**[4]												
Yes	2566	77·7	0·99 (0·85-1·16)	NA	2009	70·4	1·00 (0·86-1·16)	NA	1679	45·9	0·69 (0·57-0·83)	**0·74 (0·61-0·90)**
No	1352	77·8	1	NA	1278	70·4	1	NA	614	55·2	1	1

**Stimulant use[4,5]**												
Yes	715	76·1	0·89 (0·74-1·08)	NA	888	69·3	0·93 (0·78-1·10)	NA	140	46·4	0·92 (0·65-1·30)	NA
No	3213	78·1	1	NA	2400	70·8	1	NA	2153	48·5	1	NA

Bold: p≥0·05; OR: Odds Ratio, aOR: adjusted OR, 95%CI: 95% Confidence Interval, NA: Not Applicable. [1]MPR≥0·6 is equivalent to 4 PrEP pills per week; [2]For Brazil and Mexico: last 3 months, for Peru: last 6 months; [3]last 6 months; [4]stimulant use was defined as use of any: club drugs (e.g. ecstasy, LSD and GHB), cocaine (powder, crack, or paste), poppers or other inhalants.

Supplementary Table 4. Factors associated with long-term PrEP engagement according to country.

	Brazil				Mexico				Peru			
	N	Long-term PrEP engagement[1]	Univariable analyses	Multivariable analysis	N	Long-term PrEP engagement[1]	Univariable analyses	Multivariable analysis	N	Long-term PrEP engagement[1]	Univariable analyses	Multivariable analysis
	3854	N=3124 81·1%	OR (95%CI)	aOR (95%CI)	2434	N=1655 68·0%	OR (95%CI)	aOR (95%CI)	1937	N=1004 51·8%	OR (95%CI)	aOR (95%CI)
**Gender**												
Cisgender man	3667	82·2	1	1	2368	69·0	1	1	1702	52·8	1	1
Transgender woman	187	58·8	0·31 (0·23-0·42)	**0·48 (0·32-0·70)**	66	33·3	0·22 (0·13-0·38)	**0·26 (0·14-0·46)**	235	44·7	0·72 (0·55-0·95)	0·96 (0·68-1·36)
**Age (years)**												
18-24	1014	72·6	0·38 (0·31-0·47)	**0·51 (0·41-0·63)**	495	56·4	0·46 (0·37-0·57)	**0·55 (0·43-0·70)**	669	46·9	0·65 (0·52-0·80)	**0·63 (0·50-0·80)**
25-30	1188	80·0	0·56 (0·46-0·69)	**0·64 (0·52-0·80)**	850	67·3	0·73 (0·60-0·89)	**0·78 (0·64-0·96)**	555	50·8	0·76 (0·60-0·95)	**0·65 (0·51-0·83)**
>30	1652	87·4	1	1	1089	73·8	1	1	683	57·7	1	1
**Race**												
Black/Mix-race/Indigenous/Asian	2017	78·1	1	1	2083	67·4	1	NA	1734	52·5	1	1
White	1837	84·3	1·50 (1·27-1·77)	**1·23 (1·04-1·47)**	351	71·2	1·19 (0·93-1·53)	NA	203	46·3	0·78 (0·58-1·04)	0·78 (0·57-1·07)
**Education**												
Primary (complete or incomplete)	52	61·5	0·32 (0·18-0·56)	0·57 (0·30-1·10)	18	55·6	0·56 (0·22-1·43)	1·27 (0·44-3·64)	36	38·9	0·48 (0·24-0·94)	0·63 (0·30-1·31)
Secondary (complete or incomplete)	750	73·3	0·55 (0·46-0·66)	0·82 (0·66-1·01)	131	53·4	0·52 (0·36-0·74)	0·88 (0·59-1·30)	634	42·1	0·55 (0·45-0·66)	**0·65 (0·52-0·80)**
More than secondary	3052	83·3	1	1	2285	68·9	1	1	1267	577·1	1	1
**Main reason to attend the service**												
Seeking PrEP	3704	81·4	1·68 (1·14-2·38)	1·38 (0·93-2·05)	2337	68·1	1·15 (0·75-1·76)	NA	1133	57·1	1·67 (1·39-2·00)	**1·45 (1·18-1·76)**
Other	150	72·7	1	1	97	65·0	1	NA	804	44·4	1	1
**Number of sex partners[3]**												
< 5	1781	79·1	1	1	825	62·3	1	1	998	51·2	1	NA
5-10	953	83·7	1·36 (1·10-1·67)	**1·27 (1·02-1·58)**	830	68·0	1·28 (1·05-1·57)	**1·32 (1·06-1·63)**	501	55·1	1·17 (0·94-1·45)	NA
> 10	1120	81·9	1·19 (0·99-1·44)	**1·55 (1·24-1·94)**	779	74·1	1·73 (1·40-2·14)	**1·96 (1·54-2·50)**	438	49·5	0·94 (0·75-1·17)	NA
**Receptive CAS[3]**												
Yes	2513	81·8	1·14 (0·96-1·35)	NA	1661	68·5	1·07 (0·90-1·29)	NA	1139	53·7	1·20 (1·00-1·44)	**1·40 (1·15-1·72)**
No	1341	79·7	1	NA	773	66·9	1	NA	798	49·1	1	1
**CAS with partner(s) living with HIV[3]**												
Yes	812	84·1	1·19 (0·94-1·52)	NA	651	72·8	1·43 (1·12-1·82)	1·23 (0·96-1·59)	189	65·1	1·68 (1·20-2·34)	1·34 (0·93-1·91)
No	1111	81·6	1	NA	592	65·2	1	1	663	52·6	1	1
I don't know	1931	79·4	0·87 (0·72-1·05)	NA	1191	68·0	1·07 (0·87-1·32)	0·93 (0·75-1·16)	1085	49·0	0·86 (0·71-1·05)	0·84 (0·68-1·03)
**Transactional sex[3]**												
Yes	387	69·8	0·49 (0·39-0·62)	0·72 (0·53-0·99)	485	63·5	0·78 (0·63-0·96)	0·88 (0·69-1·13)	452	43·8	0·66 (0·53-0·81)	0·88 (0·68-1·13)
No	3467	82·3	1	1	1949	69·1	1	1	1485	54·3	1	1

**Binge drinking[3]**												
Yes	2520	81·2	1·03 (0·87-1·22)	NA	1458	68·4	1·05 (0·88-1·25)	NA	1428	50·7	0·84 (0·69-1·03)	NA
No	1334	80·7	1	NA	976	67·3	1	NA	509	55·0	1	NA
**Stimulant use[3,4]**												
Yes	699	80·7	0·97 (0·79-1·20)	NA	688	69·5	1·10 (0·91-1·33)	NA	123	48·0	0·85 (0·59-1·23)	NA
No	3155	81·1	1	NA	1746	67·4	1	NA	1814	52·1	1	NA
**Self-reported PrEP adherence at week 4[5]**												
Yes	2467	88·7	3·81 (3·22-4·50)	**3·31 (2·78-3·93)**	1488	77·4	3·00 (2·51-3·57)	**2·77 (2·31-3·32)**	566	74·0	3·83 (3·08-4·75)	**3·48 (2·79-4·35)**
No	1387	67·4	1	1	946	53·3	1	1	1371	42·8	1	1

Bold: p≥0·05; OR: Odds Ratio, aOR: adjusted OR, 95%CI: 95% Confidence Interval, NA: Not Applicable. [1]Attending the week 4 study visit plus two or more quarterly visits within a 52-week follow-up period. Participants were eligible to have the event long-term PrEP engagement if they had time to complete 52-weeks of follow-up; [2]For Brazil and Mexico: last 3 months, for Peru: last 6 months; [3]last 6 months; [4]stimulant use was defined as use of any: club drugs (e.g. ecstasy, LSD and GHB), cocaine (powder, crack, or paste), poppers or other inhalants; [5]Report of any missing pill in the previous 30 days.

Supplementary Table 5. Prevalence of syphilis at enrollment and syphilis incidence during study follow-up overall and stratified per country, age, and gender.

	Prevalence at enrollment % (95%CI)	Person-years (Incident cases of Syphilis)	Rate 100 person-years (95%CI)
Overall	8·8 (8·2-9·4)	7762·24 (783)	10·09 (9·40-10·82)
**Country**			
Brazil	11·4 (10·4-12·4)	3521·51 (420)	11·93 (10·84-13·12)
Mexico	5·1 (4·3-5·8)	2468·88 (200)	8·10 (7·05-9·31)
Peru	9·7 (8·4-10·9)	1771·84 (163)	9·20 (7·89-10·73)
**Age (years)**			
18-24	9·1 (7·9-10·2)	1891·81 (201)	10·62 (9·25-12·20)
25-30	9·3 (8·3-10·4)	2427·38 (234)	9·64 (8·48-10·96)
>30	8·3 (7·4-9·2)	3443·06 (348)	10·11 (9·10-11·23)
**Gender**			
Cisgender man	8·4 (7·8-9·0)	7376·21 (736)	9·98 (9·28-10·73)
Transgender woman	15·4 (12·4-18·5)	386·03 (47)	12·18 (9·15-16·20)

Supplementary Table 6. Prevalence of participants with rectal chlamydia and gonorrhea at enrollment and week 52 overall and stratified per country, gender, and age.

	Chlamydia % (95%CI)				Gonorrhoea % (95%CI)			
	**Enrollment**	**Week 52**	**OR[1]**	**p-value**	**Enrollment**	**Week 52**	**OR[1]**	**p-value**
**Overall**	11·8 (11·1-12·4)	10·0 (8·8-11·1)	0·85 (0·74-0·98)	0·025	9·2 (8·6-9·8)	6·7 (5·8-7·7)	0·76 (0·65-0·90)	0·0014
**Country**								
Brazil	10·3 (9·4-11·3)	11·3 (9·7-13·0)	1·09 (0·90-1·32)	0·39	6·5 (5·7-7·3)	6·7 (5·4-8·0)	1·03 (0·80-1·31)	0·83
Mexico	11·4 (10·2-12·6)	7·1 (5·3-8·9)	0·60 (0·44-0·81)	0·0009	13·2 (11·9-14·4)	7·8 (5·9-9·7)	0·56 (0·42-0·74)	<0·0001
Peru	14·6 (13·1-16·0)	10·3 (7·9-12·7)	0·68 (0·51-0·89)	0·0059	8·9 (7·8-10·1)	5·6 (3·8-7·4)	0·60 (0·42-0·87)	0·0071
**Gender**								
Cisgender man	11·4 (10·8-12·1)	9·6 (8·5-10·8)	0·82 (0·71-0·95)	0·0070	8·9 (8·3-9·5)	6·7 (5·7-7·6)	0·73 (0·62-0·87)	0·0003
Transgender woman	16·8 (13·6-20·0)	16·3 (9·9-22·6)	0·98 (0·59-1·62)	0·92	14·3 (11·2-17·3)	8·5 (3·7-13·2)	0·55 (0·29-1·06)	0·074
**Age (years)**								
18-24	14·2 (12·8-15·6)	13·5 (10·8-16·2)	0·94 (0·72-1·22)	0·65	12·1 (10·7-13·4)	9·5 (7·1-11·8)	0·76 (0·56-1·02)	0·069
25-30	12·2 (10·9-13·4)	9·8 (7·8-11·8)	0·76 (0·58-0·97)	0·030	9·2 (8·2-10·3)	6·6 (4·9-8·3)	0·69 (0·51-0·93)	0·016
> 30	9·9 (8·9-10·9)	8·5 (7·0-10·0)	0·84 (0·67-1·04)	0·11	7·4 (6·6-8·3)	5·6 (4·4-6·8)	0·75 (0·58-0·97)	0·029

[1]Odds Ratio for having Chlamydia or Gonorrhoea at week 52 compared to baseline using GEE model (for the overall population, the models were adjusted by country, gender, and age).

Supplementary Table 7. Prevalence of participants with hepatitis B and hepatitis C at enrollment and week 52 overall and stratified per country, gender, and age.

	Hepatitis B % (95%CI)				Hepatitis C % (95%CI)			
	Enrollment	Week 52	OR[1]	p-value	Enrollment	Week 52	OR[1]	p-value
**Overall**	0·47 (0·33-0·62)	0·42 (0·19-0·64)	0·87 (0·56-1·38)	0·56	0·44 (0·30-0·58)	1·03 (0·70-1·36)	2·09 (1·40-3·12)	0·0003
**Country**								
Brazil	0·76 (0·49-1·04)	0·67 (0·24-1·11)	0·97 (0·55-1·70)	0·91	0·34 (0·16-0·52)	0·58 (0·24-0·91)	1·69 (0·88-3·24)	0·12
Mexico	0·20 (0·04-0·36)	0·00 (0·00-0·34)[2]	0·34 (0·00-1·78)[3]	0·16	0·83 (0·51-1·16)	2·14 (1·27-3·00)	1·88 (1·03-3·46)	0·041
Peru	0·35 (0·11-0·60)	0·57 (0·16-1·46)[2]	0·74 (0·13-4·05)	0·73	0·09 (0·01-0·32)[2]	0·57 (0·16-1·46)[2]	5·55 (1·12-27·63)	0·036
**Gender**								
Cisgender man	0·47 (0·32-0·61)	0·40 (0·18-0·63)	0·92 (0·56-1·50)	0·73	0·43 (0·29-0·57)	1·05 (0·71-1·38)	2·09 (1·38-3·17)	0·0005
Transgender woman	0·58 (0·12-1·68)[2]	0·75 (0·02-4·09)[2]	0·94 (0·59-1·49)	0·80	0·58 (0·12-1·67)[2]	0·65 (0·02-3·59)[2]	0·99 (0·82-1·18)	0·88
**Age (years)**								
18-24	0·21 (0·03-0·40)	0·00 (0·00-0·55)[2]	0·52 (0·00-2·90) [3]	0·29	0·17 (0·05-0·43)[2]	0·40 (0·08-1·16)[2]	2·37 (0·53-10·62)	0·26
25-30	0·27 (0·08-0·46)	0·21 (0·03-0·77)[2]	0·64 (0·23-1·80)	0·40	0·37 (0·15-0·60)	1·43 (0·73-2·13)	3·13 (1·47-6·65)	0·0030
> 30	0·80 (0·51-1·08)	0·72 (0·30-1·15)	1·02 (0·61-1·68)	0·95	0·66 (0·40-0·92)	1·05 (0·58-1·62)	1·42 (0·89-2·27)	0·14

[1]Odds Ratio for having Hepatitis B or Hepatitis C at week 52 compared to baseline using GEE model (for the overall population, the models were adjusted by country, gender, and age); [2]Exact confidence interval; [3]Odds Ratio obtained using an exact logistic regression due to data sparseness.

Supplementary Table 8. Number of individuals that interrupted and discontinued PrEP due to adverse events.

Adverse events	PrEP interruption n (%)	PrEP discontinuation n (%)
Renal (Clearance ≤ 60mL/min)	31 (0·33)	14 (0·15)
Gastrointestinal	25 (0·26)	18 (0·19)
Other clinical reasons	31 (0·33)	20 (0·21)
Total	87 (0·92)	52 (0·55)

ImPrEP Study Group

Brazil

J· David Urbaez-Brito, Polyana d'Albuquerque, Claudio Palombo, Paulo Ricardo de Alencastro, Raquel Keiko de Luca Ito, João L· de Benedetti, Fabio V· Maria, Paula M· Luz, Lucilene Freitas, Kim Geraldo, Monica Derrico, Sandro Nazer, Tania Kristic, Renato Girade (*in memoriam*), Renato Lima, Antônio R· de Carvalho, Carla Rocha, Pedro Leite, Marcio Lessa, Marilia Santini, Daniel R· B· Bezerra, Cleo de Oliveira Souza, Jacinto Corrêa, Marcelo Alves, Carolina Souza, Camilla Portugal, Mônica dos Santos Valões, Gabriel Lima Mota, Joyce Alves Gomes, Cynthia Ferreira Lima Falcão, Fernanda Falcão Riberson, Luciano Melo, Talita Andrade Oliva, Agnaldo Moreira de Oliveira Júnior, Bruna Fonseca, Leonor Henriette de Lannoy, Ludymilla Anderson Santiago Carlos, João Paulo da Cunha, Sonia Maria de Alencastro Coracini, Thiago Oliveira Rodrigues, Emília Regina Scharf Mettrau, Kelly Vieira Meira; Heder Tavares, Ana Paula Nunes Viveiros Valeiras, Taiane Miyake Alves de Carvalho Rocha, Alex Amorim, Patrícia Sabadini, Luiz Gustavo Córdoba; Caio Gusmão, Erika Faustino, Julia Soares da Silva Hansen, Agatha Mirian Cunha, Neuza Uchiyama Nishimura, Jaime Eduardo Flygare Razo Prereira dos Santos, Aline Barnabé Cano, Willyam Magnum Telles Dias, Magô Tonhon, Tania Regina Rezende, Alex Gomes, Eloá dos Santos Rodrigues, Maria das Dores Aires Carneiro, Alexandre Castilho, Mariana Carvalho.

Mexico

Dulce Diaz-Sosa, Centli Guillén, Lorena Hernández, Rebeca Robles, Maria Elena Medina-Mora, Marcela González, Ivonne Huerta Icelo, Araczy Martinez Davalos, José Gomez Castro, Luis Obed Ocampo Valdez, Fernanda Ramírez Barajas, Verónica Ruiz González, Galileo Vargas Guadarrama, Israel Macías, Jehovani Tena Sánchez, Juan Pablo Osuna Noriega, H· Rodrigo Moheno M·, Jorge M· Bernal Ramírez, Víctor Dante Galicia Juarez, Gerardo Vizcaíno, Francisco Javier Arjona.

Peru

Gino Calvo, Silver Vargas, Oliver Elorreaga, Ximena Gutierrez, Fernando Olivos, Damaris Caviedes, Daniella Adriazola, Eduardo Juárez, Gabriela Mariño, Jazmin Qquellon, Francesca Vasquez, Jean Pierre Jiron, Sonia Flores, Karen Campos.

ImPrEP Study Sites

Brazil

Fundação de Medicina Tropical (Manaus, Amazonas), Hospital Universitário Oswaldo Cruz (Recife, Pernambuco), CEDAP – Centro Estadual Especializado em Diagnóstico, Assistência e Pesquisa (Salvador, Bahia), Hospital Dia Asa Sul (Brasília, Distrito Federal), Instituto Nacional de Infectologia Evandro Chagas, Fundação Oswaldo Cruz INI-Fiocruz (Rio de Janeiro), Hospital Municipal Rocha Maia (Rio de Janeiro), Hospital Municipal Carlos Tortelly (Niterói, Rio de Janeiro), Centro de Referência em DST/AIDS- AMDA (Campinas, São Paulo), Centro de Referência e Treinamento em DST/AIDS – CRT-SP (São Paulo), SAE DST/AIDS – CECI (São Paulo), SAE DST/AIDS – Fidélis Ribeiro (São Paulo), SAE Adulto (Santos, São Paulo), Poli Centro (Florianópolis, Santa Catarina), SAT – Sanatório Partenon (Porto Alegre, Rio Grande do Sul).

Mexico

Clínica Especializada Condesa (Cuauhtémoc, Mexico City), Fundación Unidos por un México Vivo A.C. (Cuauhtémoc, Mexico City), Comité Humanitario de Esfuerzo Compartido Contra El Sida A.C. (Guadalajara, Jalisco), Solidaridad Ed Thomas A·C· (Puerto Vallarta, Jalisco).

Peru

Centro de Referencia de Infecciones de Transmisión Sexual del Centro Materno Infantil San José (Lima), Centro de Referencia de Infecciones de Transmisión Sexual del Centro Materno Infantil Tahuantinsuyo Bajo (Lima), Centro de Referencia de Infecciones de Transmisión Sexual del Centro de Salud Alberto Barton (Callao), Centro de Referencia de Infecciones de Transmisión Sexual de Caja de Agua (Lima), Centro de Referencia de Infecciones de Transmisión Sexual del Hospital Amazónico Pucallpa (Ucayali), Centro de Referencia de Infecciones de Transmisión Sexual del Hospital La Caleta Chimbote (Ancash), Centro de Referencia de Infecciones de Transmisión Sexual del Hospital Regional Ica (Ica), Centro de Referencia de Infecciones de Transmisión Sexual del Hospital Regional Trujillo (La Libertad), Investigaciones Médicas en Salud, INMENSA (Lima), Centro de Referencia de Infecciones de Transmisión Sexual del Hospital San Juan De Dios, Pisco (Ica).
